# Low *OLFM1* and *BMP6* Expression Predicts Recurrence in Early-Stage Nonsquamous NSCLC with Pure Solid Tumor Appearance

**DOI:** 10.1158/2767-9764.CRC-25-0186

**Published:** 2025-12-18

**Authors:** Kenichi Suda, Yukihiro Yoshida, Hidenori Sato, Shuta Ohara, Akira Hamada, Masaya Yotsukura, Kouya Shiraishi, Ryuji Hamamoto, Yasutaka Chiba, Takashi Kohno, Yasushi Yatabe, Shun-ichi Watanabe, Yasuhiro Tsutani, Tetsuya Mitsudomi

**Affiliations:** 1 Division of Thoracic Surgery, Department of Surgery, Kindai University Faculty of Medicine, Osaka-Sayama, Japan.; 2 Department of Thoracic Surgery, National Cancer Center Hospital, Chuo-ku, Japan.; 3 Division of Multi-Omics Research, Yamagata University Well-Being Institute, Yamagata, Japan.; 4 Division of Genome Biology, National Cancer Center Research Institute, Chuo-ku, Japan.; 5 Division of Medical AI Research and Development, National Cancer Center Research Institute, Chuo-ku, Japan.; 6 Division of Biostatics, Clinical Research Center, Kindai University Hospital, Osaka-Sayama, Japan.; 7 Department of Pathology, National Cancer Center Hospital, Chuo-ku, Japan.; 8 Kindai Hospital Global Research Alliance Center, Kindai University Hospital, Osaka-Sayama, Japan.; 9 Izumi City General Hospital, Izumi, Japan.

## Abstract

Although pathologic stage I (pStage I) non–small cell lung cancer (NSCLC) has a good prognosis, some patients experience disease recurrence. Identification of prognostic markers for pStage I NSCLC may facilitate personalized perioperative treatment by expanding the candidates for adjuvant treatment. Patients with NSCLC with ground-glass opacity have excellent survival outcomes. Therefore, in this study, we explored prognostic biomarkers in pure solid nonsquamous NSCLC. We focused on nonsquamous NSCLC because the gene expression status is distinct from that of squamous cell carcinomas. RNA sequencing was performed on frozen tumor specimens from 33 patients with nonsquamous NSCLC with disease recurrence (recurrence group) and 33 counterparts (control group) extracted from patients without disease recurrence using propensity score matching (cohort 1). The candidate genes were further refined using an independent real-world cohort 2a (*N* = 125) and validated in The Cancer Genome Atlas cohort. Through the analysis of cohort 1 and cohort 2a, we found that low expression of six genes (*BMP6*, *KCNK3*, *NFASC*, *OLFM1*, *PEG3*, and *TNXB*) was associated with disease recurrence. The prognostic impact of the six genes was confirmed using The Cancer Genome Atlas lung adenocarcinoma database. Multivariable proportional hazard analysis using the cohort 2a dataset revealed that the combination of *BMP6* and *OLFM1* status predicted recurrence-free survival. In conclusion, we found that low *BMP6*/*OLFM1* gene status is a potential biomarker to identify patients with high-risk pStage I pure solid nonsquamous NSCLC after pulmonary resection.

**Significance:**

The identification of subgroups with a high risk of disease recurrence among patients with NSCLC with pStage I is an important clinical issue. We explored genetic factors in patients with pStage I NSCLC with a high-risk clinical feature and identified low expression of *OLFM1* and *BMP6* genes as candidate biomarkers. Clinical application of these biomarkers, together with others, will open an opportunity to administer adequate adjuvant treatment for these high-risk patients.

## Introduction

Complete surgical resection is the cornerstone of treatment for early-stage non–small cell lung cancer (NSCLC). Although adjuvant platinum doublet is administered to patients with pathologic stage (pStage) II to III disease, the National Comprehensive Cancer Network guidelines do not recommend such adjuvant chemotherapy for pStage IA patients (1). The Japanese guidelines also do not recommend the use of adjuvant platinum doublet for patients with pStage I NSCLC and instead recommend oral tegafur uracil (1–2 years) for pStage I NSCLC with tumor sizes larger than 2 cm. Although a good prognosis is expected for patients with pStage I NSCLC, some patients experience disease recurrence after complete surgical resection.

The great success of recent clinical trials on molecular-targeted drugs (2, 3) and immune checkpoint inhibitors (4–6) in adjuvant settings have led to the approvals of these agents as adjuvant treatments for patients with pStage II to III (IB–III for some drugs) NSCLC after pulmonary resection. However, it is not realistic to use these new drugs in all patients with pStage I NSCLC from the standpoints of cost-effectiveness and drug side effects, because the large percentage of these patients is cured by surgery alone. Therefore, the establishment of poor prognostic biomarkers in pStage I NSCLC may help identify candidates for adjuvant treatments, thereby improving the outcomes of patients with early-stage NSCLC after surgical resection.

Numerous studies have investigated the poor prognostic factors in patients with early-stage NSCLC. One of the easiest prognostic factors for clinical application in stage I nonsquamous NSCLC is the absence of ground-glass opacity (GGO) in preoperative CT imaging (7–9). Pathologic factors such as vascular/lymphatic invasion and/or high histologic grade are also reported as poor prognostic factors (10, 11). Mutational status of driver genes (12–14) and tumor suppressor genes (15, 16) is also reportedly prognostic; however, the magnitude of prognostic differences is not large enough to affect clinical decision-making.

Gene expression status is another potential prognostic biomarker for NSCLC although the results vary among studies. One potential obstacle in using gene expression status as a biomarker is the variation in gene expression by tumor histology. For example, genetic alterations in lung squamous cell carcinoma resemble alterations in squamous cell carcinomas of other organs rather than nonsquamous NSCLCs (17). Additionally, the prognostic implication of gene expression can vary by tumor stage. For example, TGFβ expression has different prognostic impacts in early-stage NSCLC versus advanced-stage disease (18).

Therefore, in this study, we aimed to identify genetic biomarkers associated with a high risk of disease recurrence by investigating a cohort with relatively homogeneous gene expression status, namely patients with pStage I nonsquamous NSCLC without GGO.

## Materials and Methods

### Patient selection and tumor samples

**Figure 1.**
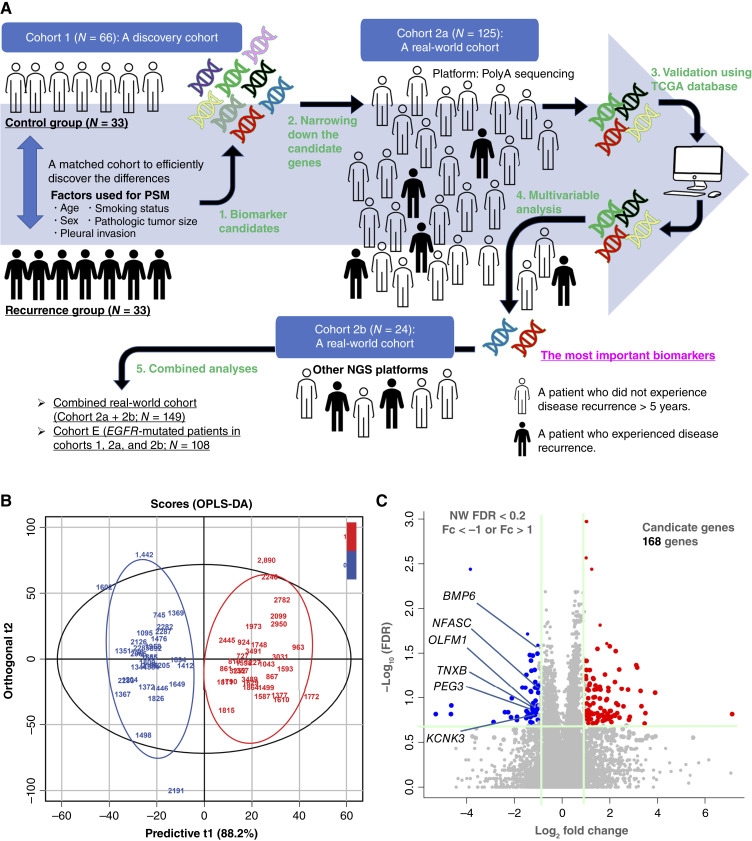
The study concept and the identification of candidate genes. **A,** The analytic flow of this study including cohort 1 (a discovery set of matched patients who experienced disease recurrence and patients who did not for >5 years), cohort 2a (a testing set of a real-world cohort), cohort 2b (additional real-world cohort), and TCGA database (a validation set). **B,** OPLS-DA analysis revealed that gene expression status differed between patients who experienced disease recurrence (red) and those who did not for >5 years (blue). **C,** A volcano plot to identify differentially expressed genes between patients who experienced disease recurrence and those who did not for >5 years. Of the genes that meet the selection criteria, upregulated or downregulated genes in the recurrence group are shown in red or blue, respectively. Fc, fold change.

This study included two independent patient cohorts: the discovery cohort (cohort 1) and the validation cohort (cohort 2; Fig. 1A). Cohort 1 was established as follows: Between January 2013 and June 2021, 942 patients received lobectomy plus mediastinal lymph node dissection for pStage I (ninth edition of tumor–node–metastasis classification) nonsquamous NSCLC at Kindai University Faculty of Medicine. Among these 942 patients, 145 patients with pure solid nodules at preoperative CT were initially recruited to this study, including 44 patients who experienced disease recurrence and 101 patients without disease recurrence and who received follow-up for 5 years or more. Among the 44 patients who experienced disease recurrence, 33 had available frozen tumor specimens for molecular analysis; these were included as the recurrence group in cohort 1 (Fig. 1A). To establish a control group for cohort 1, 1:1 propensity score matching was performed to extract 33 matched patients from the 101 patients who did not experience disease recurrence. Factors used for propensity score matching were age, sex, smoking status, pathologic tumor size, and the presence/absence of pleural invasion. Patient characteristics were similar in the two groups (Supplementary Table S1). We performed RNA sequencing (described below) on frozen tumor specimens from patients in the recurrence group (*N* = 33) and in the matched control group (*N* = 33).

Cohort 2 included patients with pStage I nonsquamous NSCLC who received lobectomy plus mediastinal lymph node dissection at the National Cancer Center (NCC) Hospital. These patients were selected from those who were enrolled in the PRISM project, in which RNA sequencing of the primary tumor tissue was prospectively performed (19, 20). The inclusion criteria were pulmonary resection before July 2018, R0 resection, pure solid tumor at preoperative CT examination, and follow-up period of 5 years or more for patients without disease recurrence. Exclusion criteria were tumors with neuroendocrine or pleomorphic component and patients with multiple lung cancers or advanced-stage tumors in other organs. A total of 149 patients were selected for cohort 2. Mutational status and expression data for the genes identified in the analysis of cohort 1 and anonymized patient data were obtained from the PRISM database for these 149 patients.

For cohort E (*EGFR*-mutated cohort), we extracted patients with activating *EGFR* mutations from cohort 1 and cohort 2. A total of 108 patients were included in cohort E.

This study adhered to the Declaration of Helsinki and was conducted according to protocols approved by the institutional review boards of Kindai University Faculty of Medicine (no. R04-078) and NCC Hospital (no. 2022-119). In this study, we analyzed tumor specimens collected under a separate research protocol that was approved by the institutional review board of Kindai University Faculty of Medicine (no. 24-071). At the time of specimen collection, written informed consent was obtained from all patients. For patients currently attending our hospital, written informed consent was obtained again for use in this study as well, and for patients who cannot provide the second informed consent because of discontinuation of treatment at our hospital, informed consent was obtained through an opt-out method. Cohort 2 patient samples were obtained from the NCC Biobank and the RNA sequencing analysis for this cohort was approved by the Institutional Review Board (IORG0002238) of the NCC (no. 2005-109). This study has been registered at Japan Registry of Clinical Trials (jRCT1050220114).

### RNA sequencing

Total RNA was extracted from frozen tissue specimens obtained from the 66 patients in cohort 1 using the RNeasy Mini Kit (Qiagen, cat. #74104). The quality of RNA was assessed by the RNA integrity number score.

RNA sequencing was performed at the Division of Multiomics research, Yamagata University Well-being Institute. The cDNA sequence library was constructed from 1 μg of rRNA-depleted RNA using the Ion Total RNA-Seq Kit v2 (Thermo Fisher Scientific, cat. #4479789) following the manufacturer’s protocol. Library quantification was performed on a 4150 TapeStation system using a D1000 ScreenTape (Agilent Technologies, cat. #5067-5582). Massive parallel semiconductor sequencing was performed on an Ion GeneStudio S5 XL system for 520 cycles using the Ion 540 Kit-OT2 and the Ion 540 Chip Kit (Thermo Fisher Scientific, cat. #A27766). More than 40 million reads were obtained and subjected to subsequent analysis.

### Gene expression analysis

Sequencing reads were computed using an in-house nextflow-based workflow pipeline on High Performance Computing (total 360-core CPU, total 3.85TB RAM, 10-nodes, and Linux OS) under the SLURM job scheduler management system. Sequencing reads data were preprocessed to correct base pair errors and filter shorter reads <70 bp and mapped to the human genome reference sequence (GRCh38/hg38) using the STAR program (RRID: SCR_004463). RefSeq gene-level transcript quantification and transcripts per million (TPM) calculation were performed using the RSEM bioconductor package (RRID: SCR_000262) in the R statistical program. Finally, data for 15,129 genes were obtained.

To evaluate the 15,129 transcripts in the recurrence and nonrecurrence groups, we analyzed TPM expression data to compare the cluster of expression patterns by the orthogonal partial least squares using the ropls bioconductor package (RRID: SCR_016888) for discriminant binary outcome with sevenfold cross-validation and 20 permutations. Significant levels were set at 0.05 in adjusted *P* and *Q* values. To investigate differentially expressed genes, a Mann–Whitney U test was performed on TPM expression data of each gene; significant levels were set as the fold change |log (recurrence/nonrecurrence)| > 1 and FDR <0.2. Differentially expressed genes in the recurrence group and control group were analyzed for expression patterns in the significant gene set using the nonnegative matrix factorization [k (italic)-rank = 2] algorithm in nmf packages (RRID: SCR_024284) in the R program.

### Gene mutation analysis

To analyze somatic functional variants, sequencing reads were computed using another in-house nextflow pipeline on the High Performance Computing. Sequencing reads were preprocessed as above and then mapped to the reference sequence using BWA (RRID: SCR_010910) and Bowtie2 (RRID: SCR_016368) program. Variant calling was conducted on data to determine a variant in the setting >0.2 allele fraction and 20 or more read counts using the Freebayes program (RRID: SCR_010761). After variant calling, high-confidence somatic variants were selected from gnomAD (RRID: SCR_014964) population frequencies in <0.0001 and unregistered SNPs in the Single Nucleotide Polymorphism Database or unregistered gnomAD database and registered SNPs. Functional somatic variants as predicted deleterious mutations were selected from the type of variants excluding synonymous, intergenic, or intronic variants.

To analyze the mutation spectrum, all functional somatic variants were analyzed for the mutational signature on an SBS signature database using the maftools bioconductor package (RRID: SCR_024519). A machine learning–based gene selection analysis was conducted using a hybrid method, integrating a random forest–based recursive feature elimination algorithm and a partial least square algorithm. This was performed using the caret package (RRID: SCR_021138) in the R programing language to identify candidate genes associated with recurrence and nonrecurrence.

### Statistical analysis

Recurrence-free survival (RFS) was defined as the interval between the date of pulmonary resection and the date of recurrence or death by any cause. Overall survival (OS) was defined as the interval between the date of pulmonary resection and the date of death by any cause. RFS and OS were estimated by the Kaplan–Meier method and compared using the log-rank test. Univariable and multivariable Cox regression analyses for associations between clinicopathologic parameters/biomarker status and RFS were performed. The Cochran–Armitage trend test was applied to compare clinicopathologic characteristics between subgroups divided by gene expression status.

All statistical analyses were performed with EZR (Saitama Medical Center, Jichi Medical University; ref. 21), which is a graphical user interface for R (The R Foundation for Statistical Computing). EZR is a modified version of R commander designed to add statistical functions frequently used in biostatistics.

## Results

### Gene expression analysis in cohort 1

The clinicopathologic characteristics of the 66 patients in cohort 1, including 33 patients in the recurrence group and 33 matched patients in the control group, are summarized in Supplementary Table S1. Through the gene expression analysis of RNA sequencing data from samples of cohort 1, data for 15,129 genes were obtained and processed as described in the “Materials and Methods” section. First, we performed OPLS-DA analysis using RNA sequencing data of the 66 patients. As shown in Fig. 1B, OPLS-DA scores of patients who experienced disease recurrence were clearly separated from those of the control group. This suggests that gene expression status may be a useful tool to identify patients with a higher risk of disease recurrence in pStage I NSCLCs with pure solid appearance. We next screened candidate genes with prognostic impacts using volcano plots. Using the criteria of NM FDR <0.2 and fold changes of >2.0 or <0.5, we extracted 168 candidate genes (Fig. 1C). Nonnegative matrix factorization analysis was performed using the 168 candidate genes (Supplementary Fig. S1). The candidate genes were further narrowed down to 101 genes, including 44 genes with higher expression in the recurrence group (clustered in R1, R2, and R3) and 57 genes with lower expression in the recurrence group (clustered in N1, N2, and N3). The 101 genes are listed in Supplementary Table S2.

We next evaluated the function of 101 genes included in each gene set by functional analysis using Gene Ontology enrichment and pathway analyses. Genes in N1/N2/N3 (with lower expression in the recurrence group) were associated with potential cancer-related pathways, such as NK cell–mediated cytotoxicity, DNA replication–dependent chromatin assembly, and regulation of vascular permeability, whereas genes in R1/R2/R3 (with higher expression in the recurrence group) were less associated with cancer biology (Supplementary Fig. S2). Therefore, we hypothesized that genes with downregulated expression in the recurrence group rather than those with upregulated expression may be useful prognostic biomarkers.

### Analysis of gene mutational status in cohort 1 and cohort 2

Mutational data were compared between the 33 patients in the recurrence group and 32 patients in the control group (after excluding one patient from the control group because of high sequence noise). We first performed mutational signature analysis by comparing the two groups; however, we did not observe differences between them [e.g., defective DNA mismatch repair (SBS26) was found in both groups, Supplementary Fig. S3].

We then compared the frequencies of mutated genes between the two groups (Supplementary Fig. S4). We found that three genes were more frequently mutated in the recurrence group, *SYNE1* (recurrence group: 12% vs. control group: 0%), *MACF1*, (15% vs. 3%), and *DCAF8L2* (21% vs. 6%), whereas two genes were frequently mutated in the control group, *HLA-F* (recurrence group: 12% vs. control group: 34%) and *RPL10* (6% vs. 22%). However, analysis in the validation cohort (cohort 2) revealed no prognostic impact of the mutation status of these five genes. Therefore, we focused on gene expression status rather than gene mutation status as a candidate biomarker to predict disease recurrence in pStage I NSCLCs with pure solid appearance.

### Prognostic impacts of candidate genes in cohort 2

Cohort 2 included 149 patients with pStage I nonsquamous NSCLC who met the inclusion and exclusion criteria described in the Methods (median follow-up of 76.9 months, Supplementary Table S1). PolyA sequencing had been performed in 125 patients (the clinicopathologic characteristics of these patients are summarized in Supplementary Table S3) and other RNA sequencing (ribo-zero NGS or SMART-seq) had been performed in 24 patients. Because the distribution of gene expression differed between these two groups (e.g., expression status of GJB6, Supplementary Fig. S5), we focused on the data of the 125 patients whose tumors were analyzed using PolyA sequencing (designated as cohort 2a, Fig. 1A) in the following analyses.

Among the 101 candidate genes identified in the analysis of cohort 1 (Supplementary Table S1), the expression status of six genes, bone morphogenetic protein 6 (*BMP6*), potassium two pore domain channel subfamily K member 3 (*KCNK3*), neurofascin (*NFASC*), olfactomedin 1 (*OLFM1*), paternally expressed 3 (*PEG3*), and tenascin XB (*TNXB*), was associated with disease status (recurrence or disease-free survival for at least 5 years) in cohort 2a. Lower expression of these six genes was associated with disease recurrence in both cohort 1 and cohort 2a. We thus next evaluated the prognostic impact of these six genes in lung adenocarcinoma using the Human Protein Atlas (proteinatlas.org; refs. 22, 23), which contains RNA expression and survival data of The Cancer Genome Atlas (TCGA; ref. 24). We observed that lower expression of these six genes was associated with poorer survival in the TCGA adenocarcinoma cohort; only *BMP6* expression did not show statistical significance (Supplementary Fig. S6).

### Survival analysis of cohort 2a by expression status of the six genes

**Figure 2.**
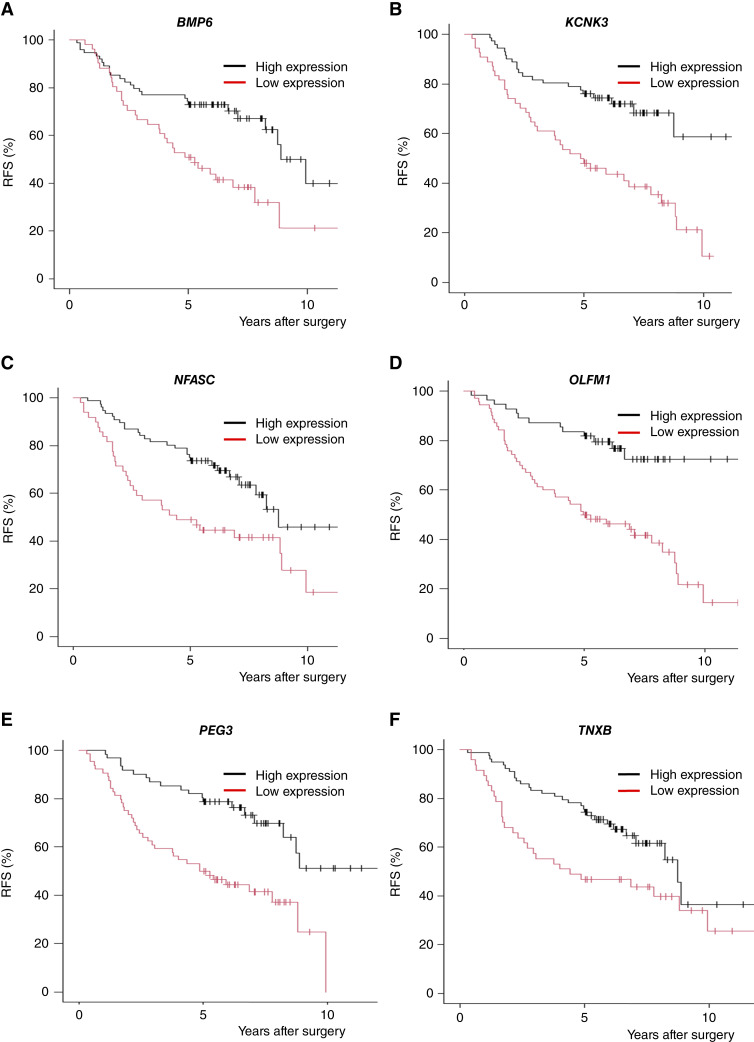
Recurrence-free survival of patients in Cohort 2a in the high expression group and low expression group for each gene (**A,** *BMP6*; **B,** *KCNK3*; **C,** *NFASC*; **D,** *OLFM1*; **E,** *PEG3*; and **F,** *TNXB*).

We then evaluated prognostic implication of the expression status of the six genes on RFS in cohort 2a (Fig. 2). For all six genes, patients with lower gene expression had worse RFS compared with patients with higher gene expression. We next analyzed the correlations of expression status of the six genes. As shown in Supplementary Fig. S7, the expression status of *BMP6* was not concordant with that of the other five genes; however, the expression status of the other five genes was associated with each other with high concordance rates (57.6%–75.2%). Furthermore, multivariable analysis of the expression status of the six genes revealed that *BMP6* and *OLFM1* expressions were independent prognostic factors for RFS (Supplementary Table S4). These results indicate that the expressions of *BMP6* and *OLFM1* were candidate prognostic markers. Prognostic implications of *BMP6* and *OLFM1* expression status were further validated using the TCGA validation cohort from the Human Protein Atlas (Supplementary Fig. S8). We also observed that the prognostic implications of these genes were prominent in early-stage lung adenocarcinomas compared with later-stage disease (Supplementary Fig. S9).

### Combination of *OLFM1* and *BMP6* expression status as a prognostic factor

**Figure 3.**
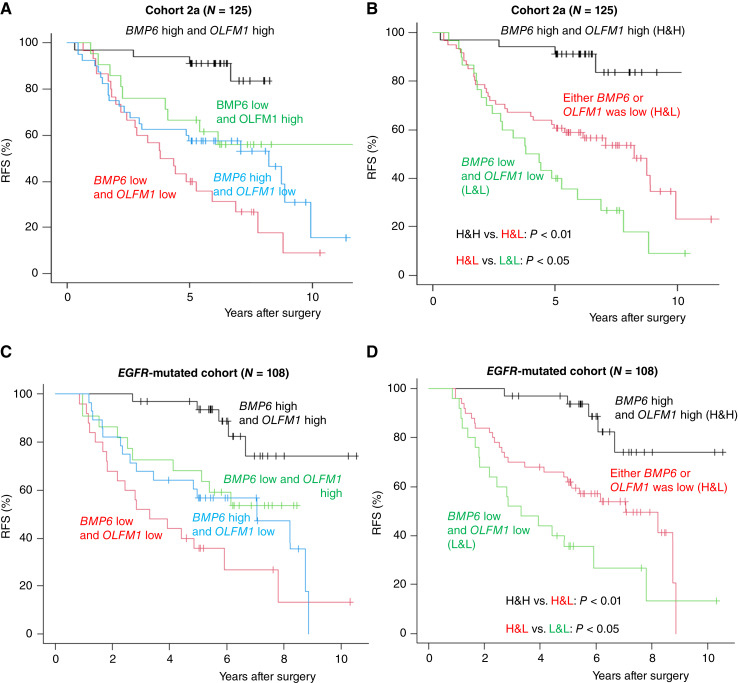
RFS of patients categorized by *BMP6* and *OLFM1* expression status. **A** and **B,** RFS in cohort 2a, a real-world cohort of patients with pStage I nonsquamous NSCLC whose tumors were analyzed using PolyA sequencing. **C** and **D,** RFS in the cohort of patients with *EGFR* mutations extracted from cohort 1 and cohort 2. H&H, high and high indicates higher expression of both *BMP6* and *OLFM1*; H&L, high and low indicates higher expression for either of the *BMP6* and *OLFM1* and lower expression for the other; L&L, low and low indicates lower expression of both *BMP6* and *OLFM1*.

We next evaluated the prognostic implications of the combinations of *OLFM1* and *BMP6* expression status on RFS. As shown in Fig. 3A and B, we found that patients with lower expression for both genes had worse prognosis, followed by patients who had lower expression for either of the two genes. Patients with higher expression of both *BMP6* and *OLFM1* showed RFS of 91.2% (95% CI, 75.1%–95.1%) at 5 years, compared with patients with lower expression of *OLFM1* or *BMP6* and patients with lower expression for both genes, who showed 5-year RFS of 60.7% [95% confidence interval (CI), 47.3%–71.6%] and 40% (95% CI, 22.8%–56.7%), respectively. We also found that the expression status of *OLFM1* and *BMP6* was associated with OS (Supplementary Fig. S10).

Multivariable analysis revealed that *OLFM1* and *BMP6* expression status was an independent prognostic factor for RFS; the HR was 4.28 (95% CI, 1.47–12.46; *P* = 0.008) in patients with low expression for either *OLFM1* or *BMP6* and 7.32 (95% CI, 2.36–22.72; *P* < 0.001) in patients with low expression for both *OLFM1* and *BMP6* compared with those who had high expression for both genes (Table 1). Similar findings were observed in multivariable analysis for OS. We also found that vascular invasion (HR, 3.13; 95% CI, 1.37–7.36; *P* = 0.007) and male sex (HR, 2.46; 95% CI, 1.19–5.06; *P* = 0.014) were independent poor prognostic factors for OS.

**Table 1.** Multivariable analysis for RFS and OS in cohort 2a (*N* = 125).

Variable	Categories	RFS			OS		
		HR	95% CI	*P* value	HR	95% CI	*P* value
*OLFM1*/*BMP6* status	1 vs. 0	4.28	1.47–12.46	0.008	2.33	0.66–8.24	0.19
	2 vs. 0	7.32	2.36–22.72	<0.001	4.18	1.13–15.50	0.032
Sex	Male vs. female	1.45	0.83–2.53	0.19	2.46	1.19–5.06	0.014
Age (years)	​	0.99	0.97–1.02	0.70	1.00	0.97–1.05	0.85
Pathologic tumor size (cm)	​	1.43	0.96–2.12	0.08	1.52	0.93–2.46	0.094
Pleural invasion	Yes vs. no	1.04	0.58–1.89	0.89	0.68	0.30–1.54	0.35
Lymphatic invasion	Yes vs. no	1.01	0.55–1.85	0.98	0.85	0.38–1.92	0.70
Vascular invasion	Yes vs. no	1.56	0.88–2.78	0.13	3.13	1.37–7.36	0.007
Adjuvant treatment	No vs. yes	1.13	0.52–2.46	0.77	1.79	0.61–5.25	0.29

We next examined the clinicopathologic characteristics of the subgroups of patients divided by *OLFM1* and *BMP6* expression status (Table 2). We observed that high-risk gene expression status was significantly associated with male sex (*P* = 0.028) and pathologic high-risk status (*P* = 0.012), which was defined as the presence of one of following factors: large invasive tumor size (>2.0 cm), pleural invasion, and/or lympho-vascular invasion.

**Table 2.** Clinicopathologic characteristics of patients divided by *OLFM1*/*BMP6* status in cohort 2a (*N* = 125) and cohort E (patients with *EGFR* mutation; *N* = 108).

Factor	High for both of *OLFM1* and *BMP6*	Low for either *OLFM1* or *BMP6*	Low for both *OLFM1* and *BMP6*	
*Cohort 2a*	(*N* = 34)	(*N* = 61)	(*N* = 30)	Trend *P* value
Sex (female/male)	27 (79%)/7 (21%)	38 (62%)/23 (38%)	16 (53%)/14 (47%)	0.028
Age (≤65/>66)	19 (56%)/15 (44%)	41 (67%)/20 (33%)	17 (57%)/13 (43%)	0.91
Smoking (yes/no)	7 (21%)/27 (79%)	24 (39%)/37 (61%)	13 (43%)/17 (57%)	0.052
Pathologic high risk (yes/no)^a^	24 (71%)/10 (29%)	53 (87%)/8 (13%)	28 (93%)/2 (7%)	0.012
Tumor size (>2 cm/≤2 cm)	20 (59%)/14 (41%)	41 (67%)/20 (33%)	21 (70%)/9 (30%)	0.34
Pleural invasion (+/−)	3 (9%)/31 (91%)	13 (21%)/48 (79%)	11 (37%)/19 (63%)	0.007
Lymphatic invasion (+/−)^a^	7 (21%)/27 (79%)	24 (39%)/37 (61%)	16 (57%)/12 (43%)	0.003
Vascular invasion (+/−)	10 (29%)/24 (71%)	35 (57%)/26 (43%)	16 (53%)/14 (47%)	0.047

*Cohort E*	(*N* = 33)	(*N* = 50)	(*N* = 25)	Trend *P* value
Sex (female/male)	28 (85%)/5 (15%)	34 (68%)/16 (32%)	17 (68%)/8 (32%)	0.13
Age (≤65/>66)	15 (56%)/18 (44%)	27 (67%)/23 (33%)	13 (57%)/12 (43%)	0.58
Smoking (yes/no)	8 (24%)/25 (76%)	13 (26%)/37 (74%)	9 (36%)/16 (64%)	0.34
Pathologic high risk (yes/no)^a^	21 (72%)/8 (28%)	42 (88%)/6 (12%)	21 (91%)/2 (9%)	0.056
Tumor size (>2 cm/≤2 cm)	16 (48%)/17 (52%)	33 (66%)/17 (34%)	14 (56%)/11 (44%)	0.48
Pleural invasion (+/−)	8 (24%)/25 (76%)	16 (32%)/34 (64%)	10 (40%)/15 (60%)	0.20
Lymphatic invasion (+/−)^a^	6 (22%)/21 (78%)	14 (31%)/31 (69%)	11 (55%)/9 (45%)	0.022
Vascular invasion (+/−)^a^	9 (33%)/18 (67%)	23 (51%)/22 (49%)	13 (59%)/9 (41%)	0.067

a Patients lacking data for lympho-vascular invasion were excluded from the analysis.

### Prognostic impacts of *OLFM1* and *BMP6* expression status in cohort 2

We further evaluated our findings using the entire cohort 2 (*N* = 149), which included patients whose tumors were analyzed using RNA sequencing platforms other than PolyA sequencing. Because it is not possible to simply compare gene expression data obtained by different platforms (Supplementary Fig. S6), we adjusted the gene expression status of tumors analyzed by platforms other than PolyA sequencing before combining with cohort 2a. Survival analysis showed that the combination of *BMP6* and *OLFM1* was also prognostic in the entire cohort 2 (Supplementary Fig. S11).

### Prognostic impacts of *OLFM1* and *BMP6* in patients with *EGFR* mutation

To evaluate the prognostic impacts of *OLFM1* and *BMP6* in patients with *EGFR* mutation, the most frequent molecular subtype of NSCLC in East Asians, we further performed an analysis combining all patients positive for *EGFR* mutation from cohort 1 and cohort 2 into cohort E. The clinicopathologic characteristics in patient groups divided by *OLFM1* and *BMP6* gene expression status are summarized in Table 2. Similar to the analysis of cohort 2a, we observed that 91% of patients with lower *OLFM1* and *BMP6* gene expression, which means high-risk gene expression status, had pathologic high-risk features compared with 72% in those with higher *OLFM1* and *BMP6* gene expression status.

In univariable and multivariable analyses, the expression status of *OLFM1* and *BMP6* was a prognostic factor for RFS and OS in the *EGFR*-positive cohort (Fig. 3C and D; Supplementary Fig. S10B; Table 3).

**Table 3.** Multivariable analysis for RFS and OS in patients with *EGFR* mutation (cohort E; *N* = 108).

Variable	Categories	RFS			OS		
		HR	95% CI	*P* value	HR	95% CI	*P* value
*OLFM1*/*BMP6* status	1 vs. 0	2.29	0.81–6.44	0.12	1.07	0.26–4.45	0.93
	2 vs. 0	7.81	2.35–25.95	<0.001	4.90	1.04–23.06	0.045
Sex	Male vs. female	1.39	0.69–2.79	0.36	1.28	0.47–3.53	0.63
Age (years)		0.96	0.93–0.99	0.014	0.95	0.90–1.00	0.063
Pathologic tumor size (cm)		1.66	0.98–2.82	0.059	0.95	0.43–2.10	0.90
Pleural invasion	Yes vs. no	1.03	0.53–2.00	0.93	0.78	0.29–2.12	0.63
Lymphatic invasion	Yes vs. no	0.68	0.31–1.49	0.33	0.57	0.16–2.01	0.38
Vascular invasion	Yes vs. no	2.22	1.11–4.43	0.024	3.26	1.02–10.45	0.047
Adjuvant treatment	No vs. yes	0.57	0.24–1.36	0.21	0.97	0.20–4.68	0.97

## Discussion

Although numerous prognostic factors have been reported in patients with NSCLC after pulmonary resection, pathologic stage is still the only determinant for the application of adjuvant treatment in many guidelines. One of the potential difficulties to identify additional prognostic biomarkers is the diversity of surgically resectable NSCLC. Therefore, in this study, we focused on pStage I nonsquamous NSCLC, and we also excluded patients with GGO who tend to show excellent survival after pulmonary resection (7–9). This strategy was useful to investigate gene expression status as a prognostic biomarker because molecular aberrations are reportedly distinct between nonsquamous NSCLCs with and without GGO component (25, 26). We found that lower expression of *OLFM1* and *BMP6* was associated with disease recurrence in three independent cohorts (cohort 1, cohort 2a/cohort 2, and TCGA database). We also observed that lower expression of *OLFM1* and *BMP6* was a poor prognostic factor in patients with pStage I nonsquamous NSCLC with *EGFR* mutation (cohort E). Although our results were consistent in these independent cohorts, as limitations of this study, we note that this study was performed using retrospective cohorts that included relatively small numbers of patients and that the biological implications of the identified biomarkers are not fully understood.

The *OLFM1* (olfactomedin 1) gene is located at 9q34.3 and is a member of the olfactomedin domain–containing protein family that encodes a protein that is abundantly expressed in brain. Although the exact function of this protein is unknown, some studies suggested that decreased *OLFM1* expression is associated with tumorigenesis, increased malignant potential, and worse progression-free survival and OS in colorectal cancer, endometrial cancer, and thyroid carcinoma (27–29). One of these studies suggested that decreased expression of *OLFM1* accelerates tumor progression through activation of the NF-κB signaling (27). The association between decreased *OLFM1* expression and activated Wnt/β-catenin signaling pathway was also reported in a study on embryo transition (30, 31).

The biological function of BMP6 (bone morphogenetic protein 6) has been clarified. The *BMP6* gene encodes a secreted ligand of the TGFβ superfamily of proteins. BMP6 binds various TGFβ receptors, leading to recruitment and activation of SMAD family transcription factors that regulate gene expression. Loss of BMP signaling, and that of its receptors, has been shown to increase WNT signaling and cancer stem cells in colorectal cancer (32). Associations between decreased *BMP6* expression and tumorigenesis, high malignant potential, and/or poorer survival were reported in patients with breast cancer, gastric cancer, and NSCLC (33–39), whereas opposite associations were reported in prostate cancer (40–42). The molecular mechanisms for decreased *OLFM1* and *BMP6* expression in high-risk nonsquamous NSCLC tumors are unclear. However, Wnt activation reportedly downregulates *OLFM1* expression in fallopian tubal epithelial cells (30), and it is possible that decreased *BMP6* expression downregulates *OLFM1* expression through activated Wnt signaling.

In this study, we found that the expression status of only two genes (*OLFM1* and *BMP6*) predicted patient outcomes after pulmonary resection. This contrasts with previous studies that identified 10 to 15 gene signatures as a prognostic biomarker in surgically resected patients with NSCLC (43–46). We consider that the exclusion of squamous cell carcinomas in this study partially contributed to the identification of these two biomarkers because analyses of the TCGA dataset suggested that six genes identified through the analyses of cohort 1 and cohort 2a did not have prognostic impact (*OLFM1* and *NFASC*) or had opposite prognostic implications (*BMP6*, *KCNK3*, *PEG3*, and *TNXB*) in squamous cell carcinomas, as shown in Supplementary Fig. S6.

Molecular-targeted drugs and immune checkpoint inhibitors, which are effective but costly treatments, are now being applied in the perioperative setting for early-stage NSCLCs. However, the risk of disease recurrence is much lower in patients with pStage I NSCLC compared with those with stage II to III disease. Therefore, it does not make sense to recommend adjuvant treatment for all patients with pStage I NSCLC but biomarker selection is definitely necessary to identify high-risk patients to maximize the cost-effectiveness of adjuvant treatments and reduce the risk of unnecessary side effects. Among patients with nonsquamous NSCLC, postoperative disease recurrences are usually seen in tumors without GGO, which often accompany pathologic high-risk features and other high-risk clinical factors. Therefore, additional biomarkers such as those found in this study may help to narrow the list of patients who benefit from adjuvant treatment. Some clinical trials of adjuvant treatment are now enrolling patients with stage I NSCLCs with pathologic high-risk features such as pleural and/or lympho-vascular invasion (47). In this study, we observed that *OLFM1* and *BMP6* expression status was significantly associated with pathologic poor prognostic factors, including lympho-vascular invasion, in both cohort 2a and cohort E (patients with *EGFR* mutation; Table 2). Multivariable analyses revealed that *OLFM1*/*BMP6* expression status was an independent prognostic factor after adjustment for these pathologic factors (Tables 1 and 3).

Another strategy to identify high-risk patients is the detection of minimal residual disease (MRD) using liquid biopsy such as ctDNA analysis. The usefulness of ctDNA analysis as a tool to stratify the risk of recurrence for adjuvant treatment has been reported in some solid tumors such as colorectal carcinomas and urothelial cancer (48, 49). However, the sensitivity of ctDNA analysis in detecting MRD is much lower in NSCLCs (50), including stage I NSCLCs with *EGFR* mutation (51). Therefore, MRD analysis is currently far from clinical application in pStage I NSCLC although preoperative ctDNA detection may indicate clinical stage I NSCLCs with high malignant potential (52).

In conclusion, through a three-step analysis, (i) exploration of candidate genes in a case–control setting, (ii) narrowing down candidate genes using a real-world cohort, and (iii) validation using TCGA data and combined cohorts, we identified that the lower expression of *OLFM1* and *BMP6* is a biomarker to identify patients with high-risk pStage I nonsquamous NSCLC with pure solid appearance at preoperative CT. We found that the genetic high-risk score was associated with pathologic high-risk features. Therefore, we consider that the combination of these factors may be useful to identify patients with high-risk stage I NSCLC who may benefit from adjuvant treatment after pulmonary resection.

## Supplementary Material

Supplementary Figure S1 Figure S1. Study One-hundred sixty eight genes which were differently expressed between these two groups were further narrowed down by NMF analysis. For the details of genes included in R1/R2/R3 and N1/N2/N3, please see Supplementary Table S1.

Supplementary Figure S2 Figure S2. Results of the Reactome Pathway Analysis in each gene set; A, B, C, D, E, and F show the results of pathway analysis (cnet) for genes included in R1. R2. R3. N1, N2, and N3, respectively.

Supplementary Figure S3 Figure S3. Mutational signature analysis in Recurrence group (A) and in Control group (B).

Supplementary Figure S4 Figure S4. List of genes of which the frequencies of mutation were different between recurrence group and control group. Green, red, and black indicate missense mutation, nonsense mutation, and multi-hits, respectively.

Supplementary Figure S5 Figure S5. Differences in gene expression levels between samples analyzed by PolyA sequencing vs. samples analyzed by other NGS (ribozero NGS or SMART-seq).

Supplementary Figure S6 Figure S6. Prognostic implications of 6 genes identified through the analysis of Cohort 1 and Cohort 2P.

Supplementary Figure S7 Figure S7. Concordance rates (A) between identified 6 genes and p-values (B).

Supplementary Figure S8 Figure S8. Prognostic implications of BMP6 and OLFM1 in TCGA variation cohort in the Human Protein Atlas.

Supplementary Figure S9 Figure S9. Prognostic implications of BMP6 and OLFM1 (stage-specific analysis).

Supplementary Figure S10 Figure S10. Overall survivals based on BMP6 and OLFM1 expression status in Cohort 2P (A) and in EGFR mutated cohort (B).

Supplementary Figure S11 Figure S11. Recurrence-free (A) and overall survivals (B) based on BMP6 and OLFM1 expression status in the entire Cohort 2.

Supplementary Table S1 Table S1. Clinical characteristics of Patients (Cohort 1 and Cohort 2)

Supplementary Table S2 Table S2. The list of differently expressed genes in Cohort 1

Supplementary Table S3 Table S3. Clinical characteristics of Patients

Supplementary Table S4 Table S4. Multivariable analysis of 6 genes for RFS in the Cohort 2a (N = 125)

## Data Availability

The data are available upon request and will be made available in the relevant repository when government services are restored.
