# Supplementary material for: Low OLFM1 and BMP6 Expression Predicts Recurrence in Early-Stage Nonsquamous NSCLC with Pure Solid Tumor Appearance
Source: Cancer Res Commun. 2025 Dec 18;5(12):2186–96. doi: 10.1158/2767-9764.CRC-25-0186 (PMC12711631; doi:10.1158/2767-9764.CRC-25-0186)
Supplement: Supplementary Figure S1 — Figure S1. Study One-hundred sixty eight genes which were differently expressed between these two groups were further narrowed down by NMF analysis. For the details of genes included in R1/R2/R3 and N1/N2/N3, please see Supplementary Table S1. [file crc-25-0186_supplementary_figure_s1_suppsf1.pdf]

Supplementary Figure S1

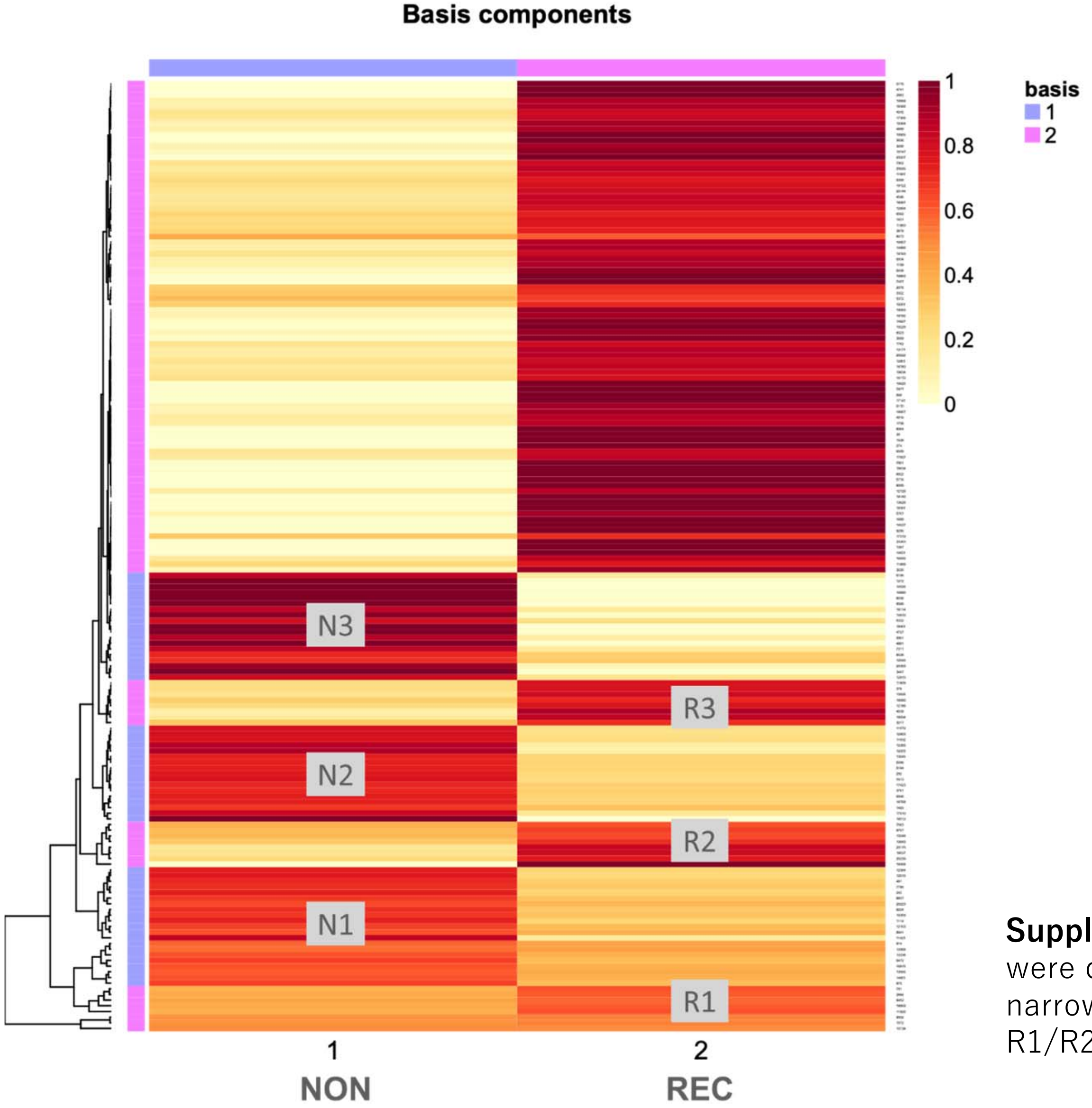

Supplementary Figure S1. Study One-hundred sixty eight genes which were differently expressed between these two groups were further narrowed down by NMF analysis. For the details of genes included in R1/R2/R3 and N1/N2/N3, please see Supplementary Table S1.
