# Supplementary material for: Low OLFM1 and BMP6 Expression Predicts Recurrence in Early-Stage Nonsquamous NSCLC with Pure Solid Tumor Appearance
Source: Cancer Res Commun. 2025 Dec 18;5(12):2186–96. doi: 10.1158/2767-9764.CRC-25-0186 (PMC12711631; doi:10.1158/2767-9764.CRC-25-0186)
Supplement: Supplementary Figure S2 — Figure S2. Results of the Reactome Pathway Analysis in each gene set; A, B, C, D, E, and F show the results of pathway analysis (cnet) for genes included in R1. R2. R3. N1, N2, and N3, respectively. [file crc-25-0186_supplementary_figure_s2_suppsf2.pdf]

Supplementary Figure S2

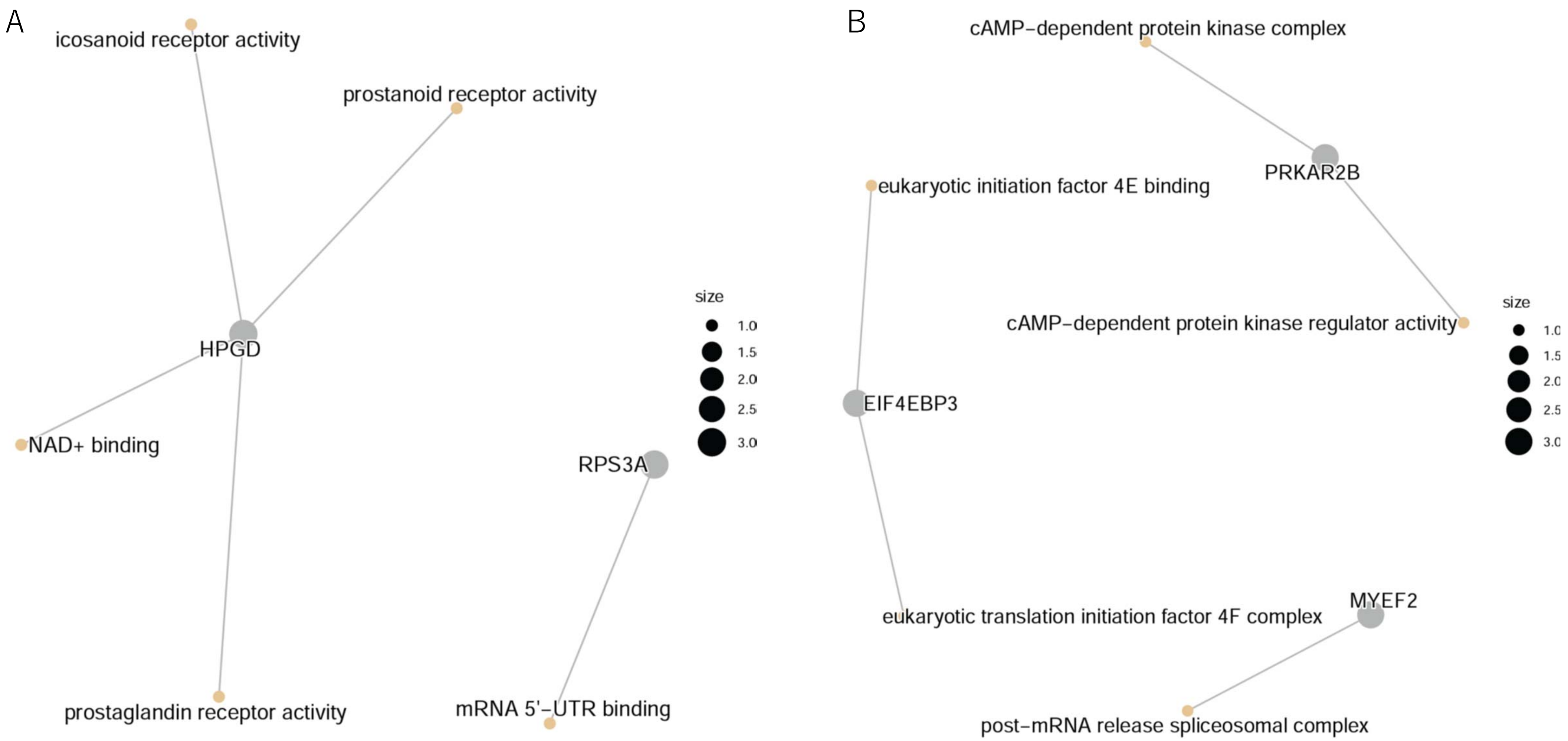

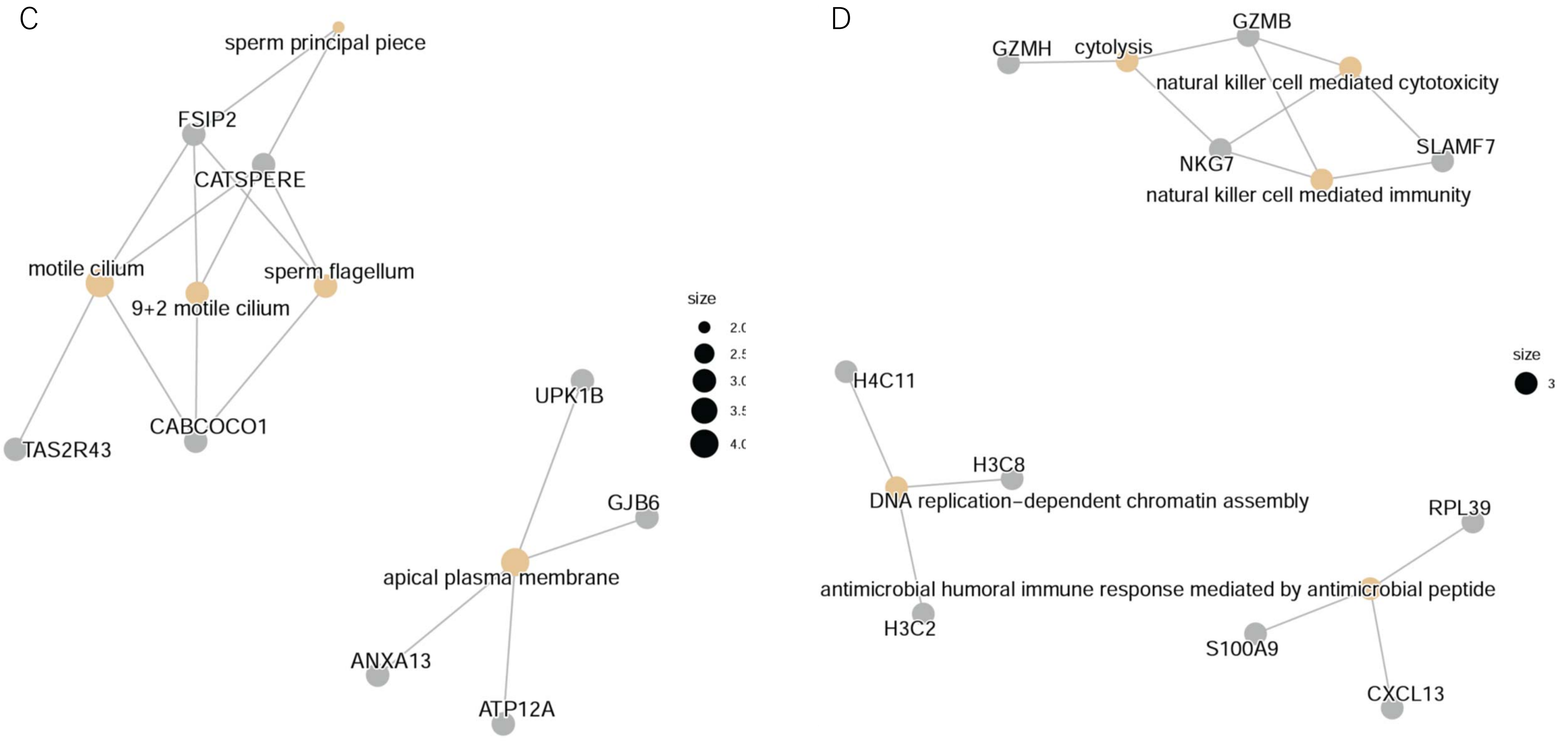
C
sperm principal piece
FSIP2
CATSPERE
motile cilium
9+2 motile cilium
sperm flagellum
TAS2R43
CABCOCO1
UPK1B
GJB6
apical plasma membrane
ANXA13
ATP12A
size
D
GZMH
cytolysis
GZMB
natural killer cell mediated cytotoxicity
NKG7
SLAMF7
natural killer cell mediated immunity
H4C11
H3C8
DNA replication−dependent chromatin assembly
RPL39
antimicrobial humoral immune response mediated by antimicrobial peptide
H3C2
S100A9
CXCL13
size
3

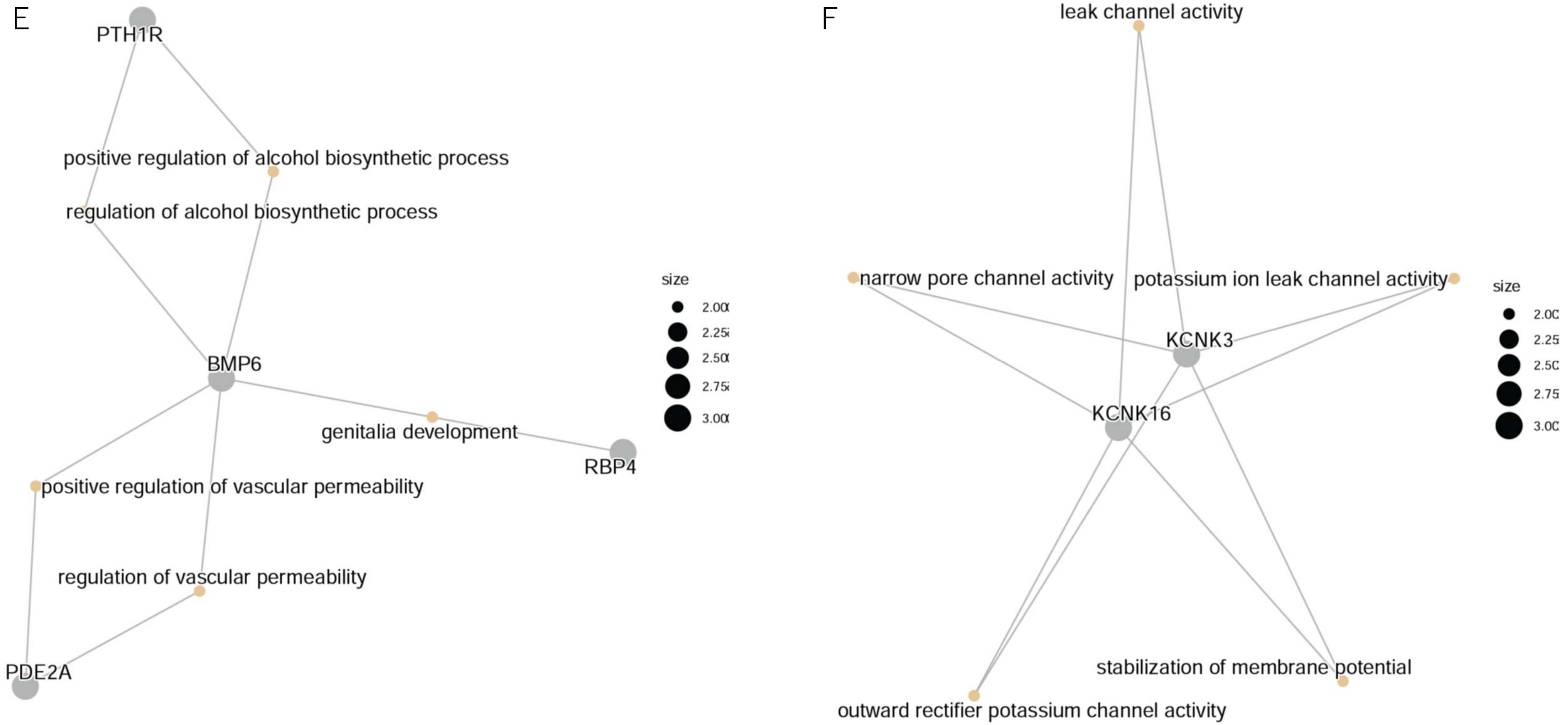

Supplementary Figure S2. Results of the Reactome Pathway Analysis in each gene set; A, B, C, D, E, and F show the results of pathway analysis (cnet) for genes included in R1. R2. R3. N1, N2, and N3, respectively.
