# Supplementary material for: Low OLFM1 and BMP6 Expression Predicts Recurrence in Early-Stage Nonsquamous NSCLC with Pure Solid Tumor Appearance
Source: Cancer Res Commun. 2025 Dec 18;5(12):2186–96. doi: 10.1158/2767-9764.CRC-25-0186 (PMC12711631; doi:10.1158/2767-9764.CRC-25-0186)
Supplement: Supplementary Figure S4 — Figure S4. List of genes of which the frequencies of mutation were different between recurrence group and control group. Green, red, and black indicate missense mutation, nonsense mutation, and multi-hits, respectively. [file crc-25-0186_supplementary_figure_s4_suppsf4.pdf]

Supplementary Figure S4

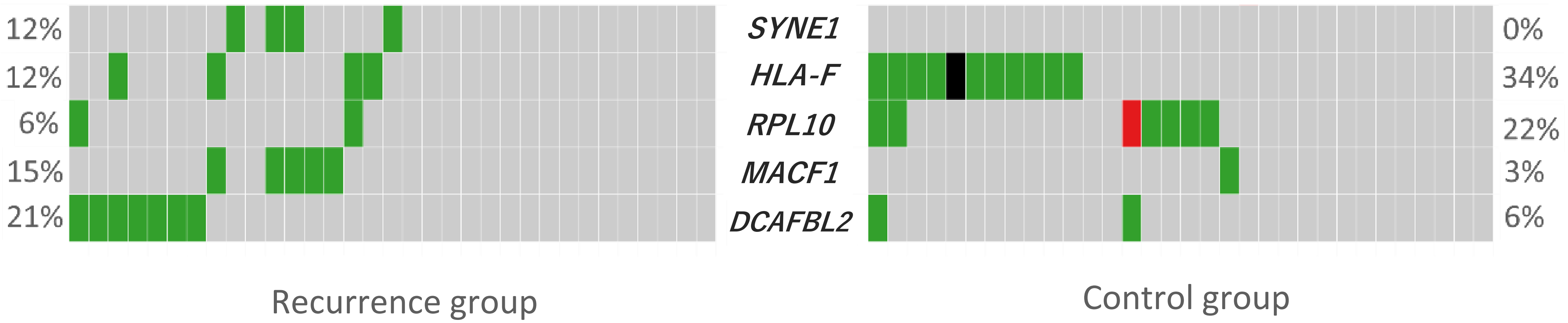

Supplementary Figure S4. List of genes of which the frequencies of mutation were different between recurrence group and control group. Green, red, and black indicate missense mutation, nonsense mutation, and multi-hits, respectively.
