# Supplementary material for: Low OLFM1 and BMP6 Expression Predicts Recurrence in Early-Stage Nonsquamous NSCLC with Pure Solid Tumor Appearance
Source: Cancer Res Commun. 2025 Dec 18;5(12):2186–96. doi: 10.1158/2767-9764.CRC-25-0186 (PMC12711631; doi:10.1158/2767-9764.CRC-25-0186)
Supplement: Supplementary Figure S5 — Figure S5. Differences in gene expression levels between samples analyzed by PolyA sequencing vs. samples analyzed by other NGS (ribozero NGS or SMART-seq). [file crc-25-0186_supplementary_figure_s5_suppsf5.pdf]

## Supplementary Figure S5

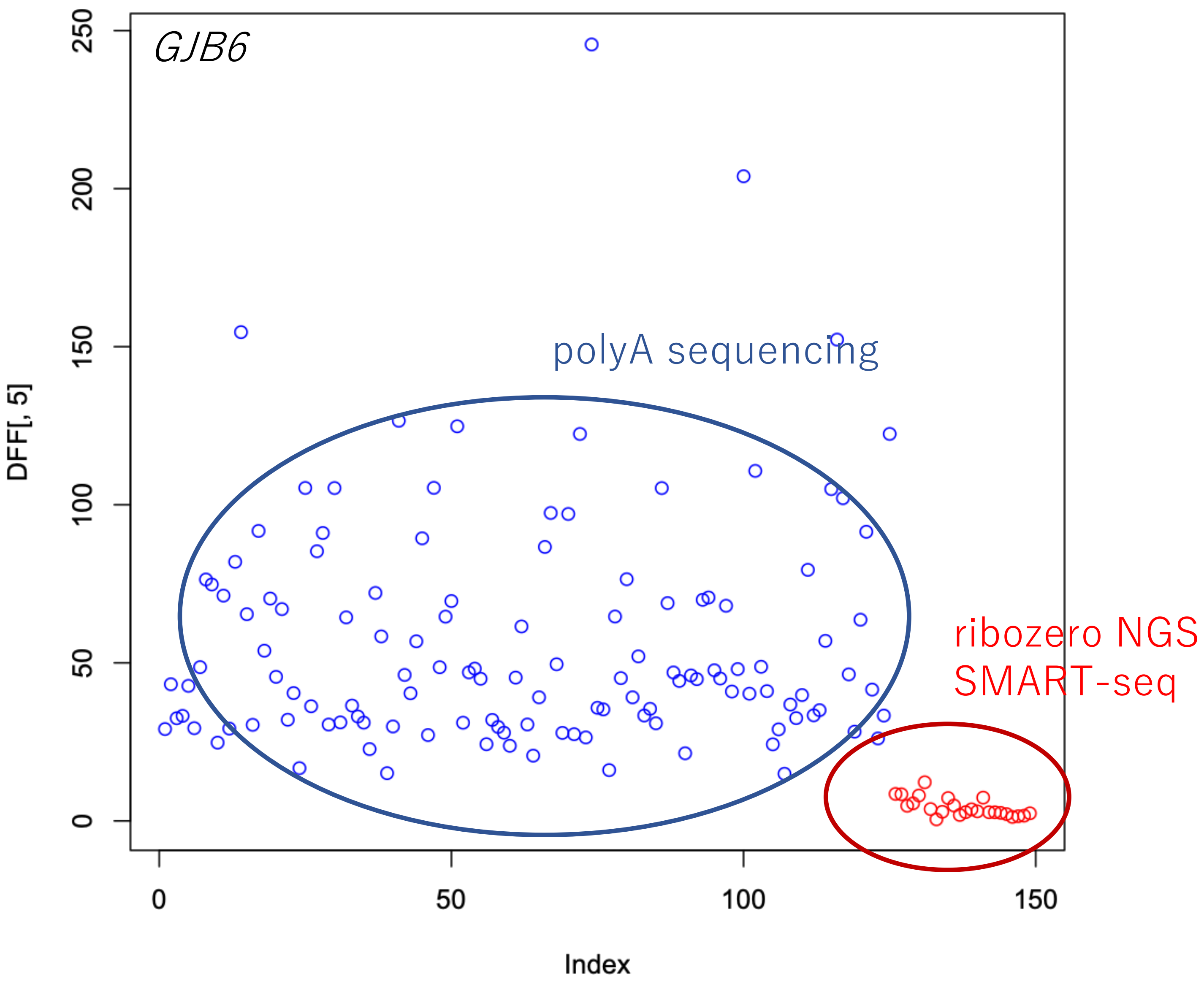


**Supplementary Figure S5.** Differences in gene expression levels between samples analyzed by PolyA sequencing vs. samples analyzed by other NGS (ribozero NGS or SMART-seq).
