# Supplementary material for: Low OLFM1 and BMP6 Expression Predicts Recurrence in Early-Stage Nonsquamous NSCLC with Pure Solid Tumor Appearance
Source: Cancer Res Commun. 2025 Dec 18;5(12):2186–96. doi: 10.1158/2767-9764.CRC-25-0186 (PMC12711631; doi:10.1158/2767-9764.CRC-25-0186)
Supplement: Supplementary Figure S6 — Figure S6. Prognostic implications of 6 genes identified through the analysis of Cohort 1 and Cohort 2P. [file crc-25-0186_supplementary_figure_s6_suppsf6.pdf]

# Supplementary Figure S6

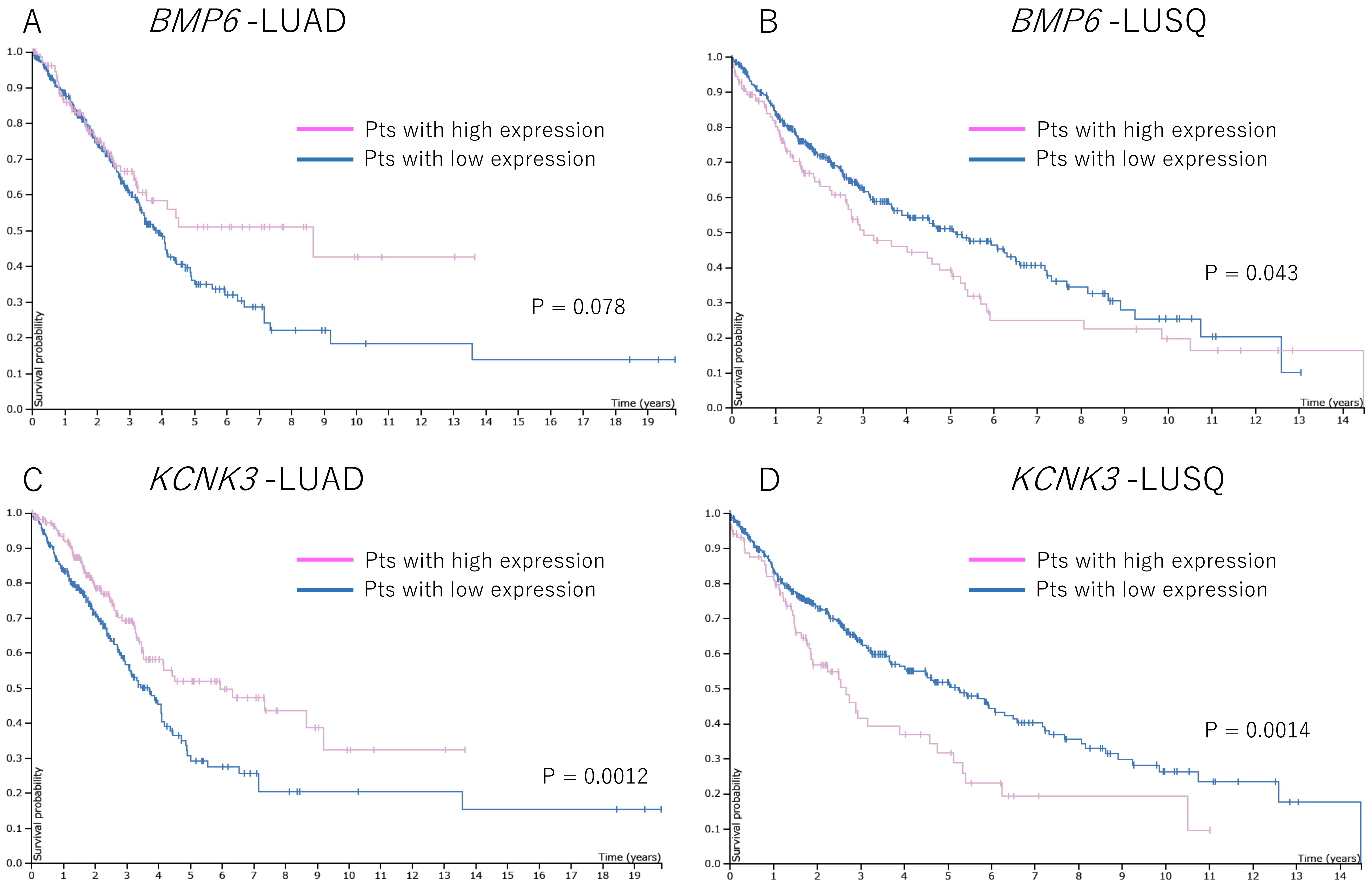

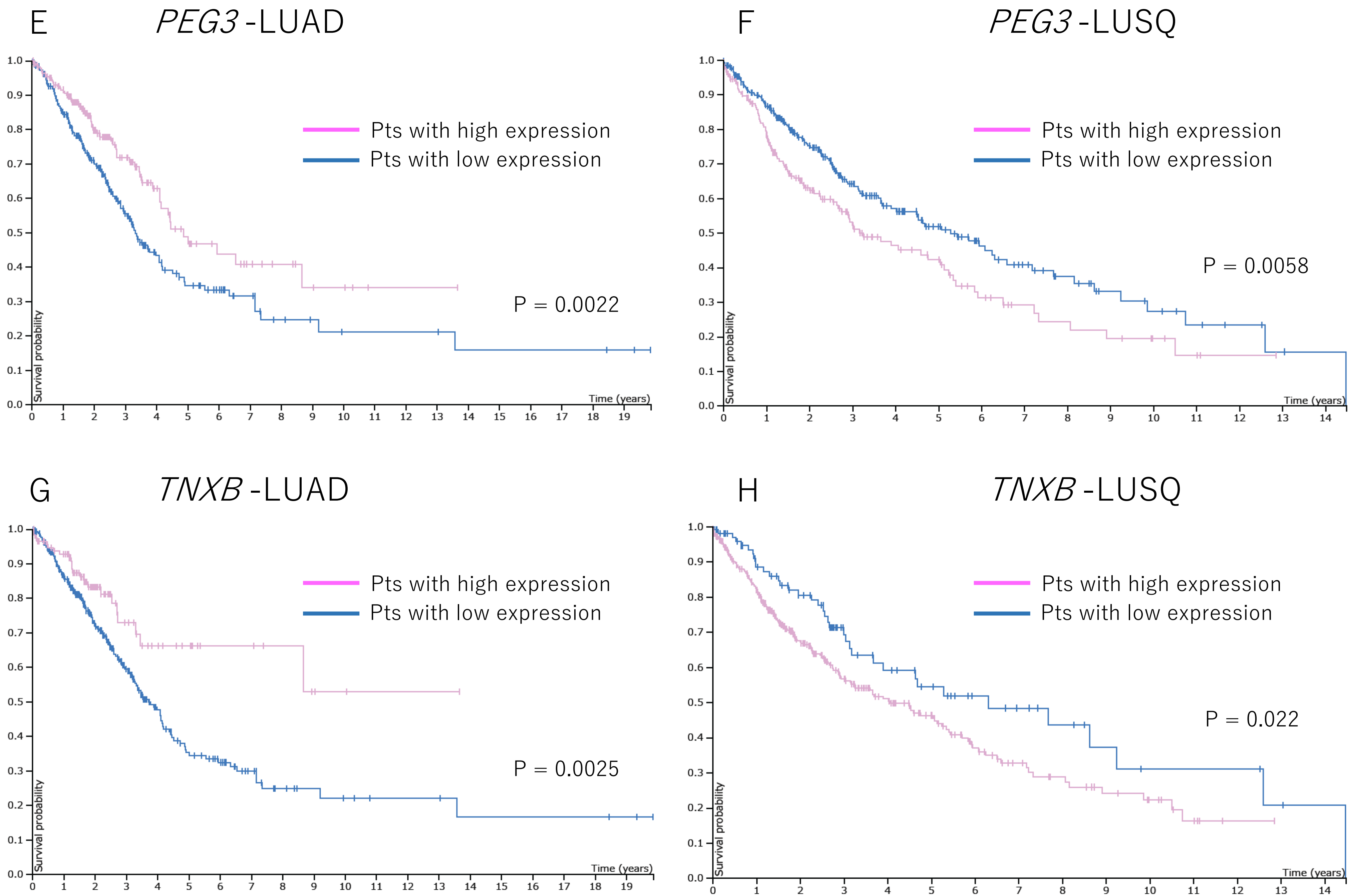

E
PEG3 -LUAD
Pts with high expression
Pts with low expression
P = 0.0022
Survival probability
Time (years)
F
PEG3 -LUSQ
Pts with high expression
Pts with low expression
P = 0.0058
Survival probability
Time (years)
G
TNXB -LUAD
Pts with high expression
Pts with low expression
P = 0.0025
Survival probability
Time (years)
H
TNXB -LUSQ
Pts with high expression
Pts with low expression
P = 0.022
Survival probability
Time (years)

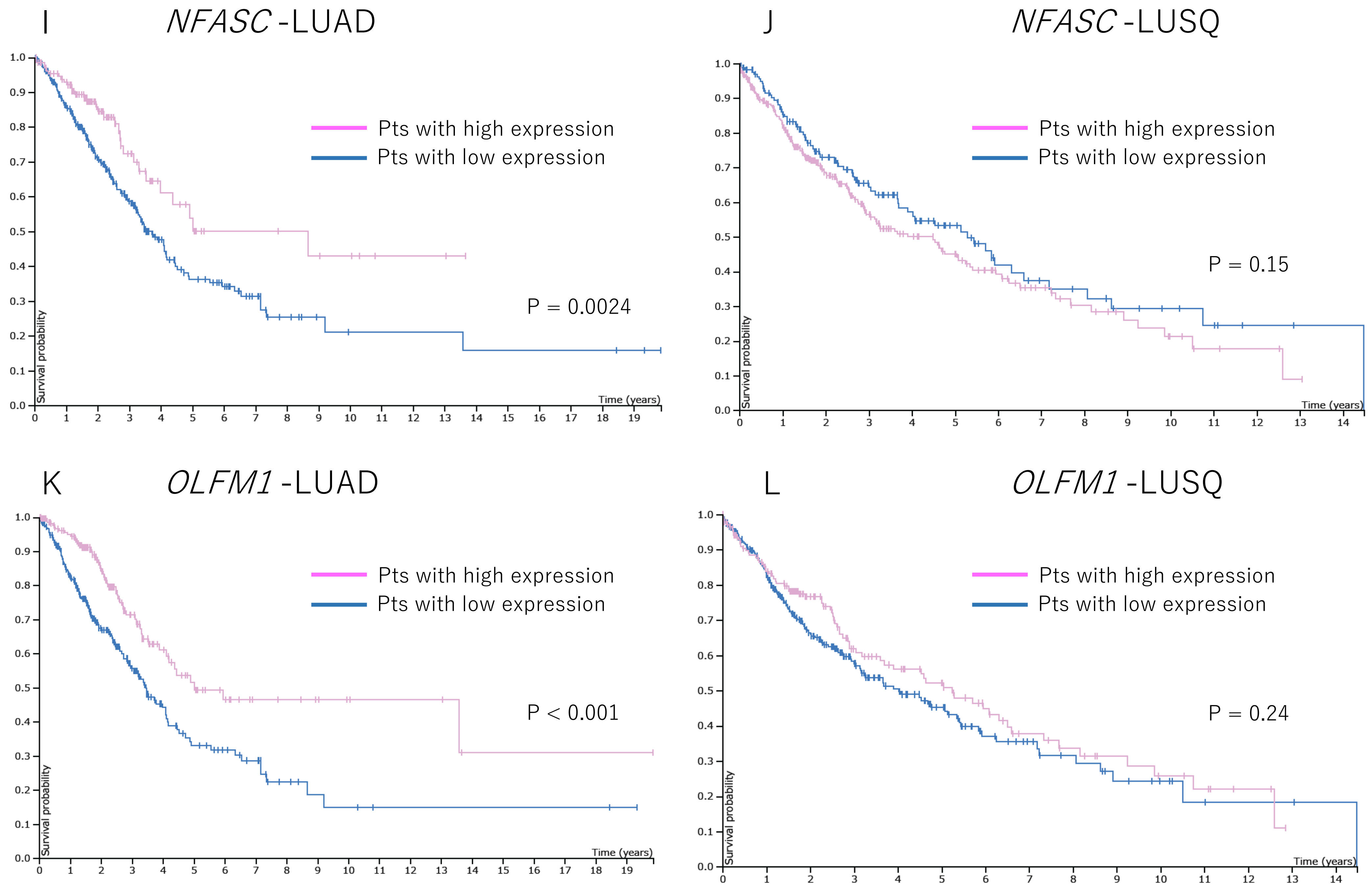


**Supplementary Figure S6.** Prognostic implications of 6 genes identified through the analysis of Cohort 1 and Cohort 2P.
