# Supplementary material for: Low OLFM1 and BMP6 Expression Predicts Recurrence in Early-Stage Nonsquamous NSCLC with Pure Solid Tumor Appearance
Source: Cancer Res Commun. 2025 Dec 18;5(12):2186–96. doi: 10.1158/2767-9764.CRC-25-0186 (PMC12711631; doi:10.1158/2767-9764.CRC-25-0186)
Supplement: Supplementary Figure S7 — Figure S7. Concordance rates (A) between identified 6 genes and p-values (B). [file crc-25-0186_supplementary_figure_s7_suppsf7.pdf]

Supplementary Figure S7

A

	BMP6	*KCNK3*	*NFASC*	*OLFM1*	*PEG3*	*TNXB*
BMP6		52.8	60.0	51.2	60.8	52
KCNK3			75.2	66.4	69.6	75.2
NFASC				57.6	69.6	74.4
OLFM1					63.2	60.8
PEG3						62.4
TNXB						

B

	BMP6	*KCNK3*	*NFASC*	*OLFM1*	*PEG3*	*TNXB*
BMP6		0.854	0.066	0.714	0.018	1
KCNK3			<0.0001	0.0001	<0.0001	<0.0001
NFASC				0.045	<0.0001	<0.0001
OLFM1					0.004	0.005
PEG3						0.005
TNXB						

Supplementary Figure S7. Concordance rates (A) between identified 6 genes and p-values (B).
