# Supplementary material for: Low OLFM1 and BMP6 Expression Predicts Recurrence in Early-Stage Nonsquamous NSCLC with Pure Solid Tumor Appearance
Source: Cancer Res Commun. 2025 Dec 18;5(12):2186–96. doi: 10.1158/2767-9764.CRC-25-0186 (PMC12711631; doi:10.1158/2767-9764.CRC-25-0186)
Supplement: Supplementary Figure S8 — Figure S8. Prognostic implications of BMP6 and OLFM1 in TCGA variation cohort in the Human Protein Atlas. [file crc-25-0186_supplementary_figure_s8_suppsf8.pdf]

Supplementary Figure S8

A *BMP6* –LUAD (validation)

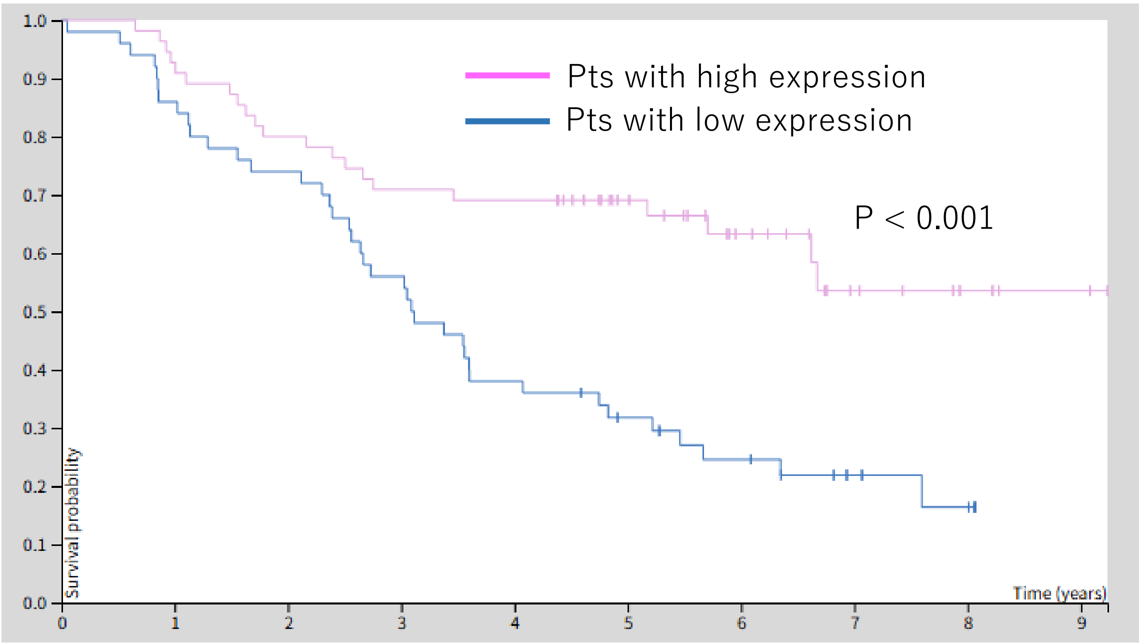

B *OLFM1* –LUAD (validation)

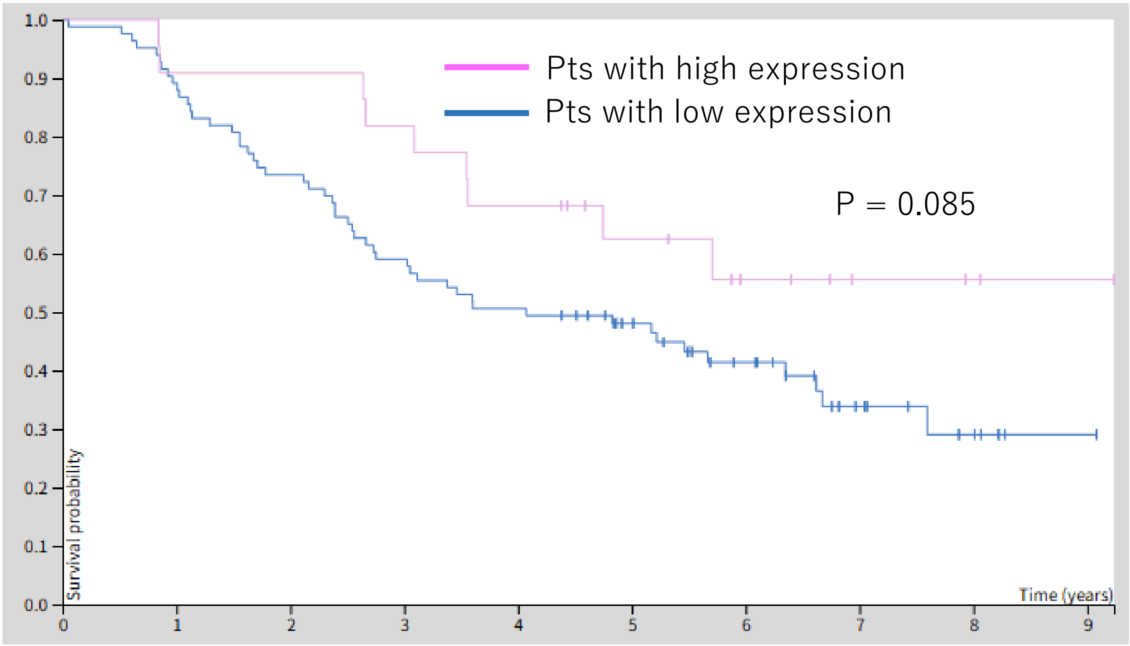

Supplementary Figure S8. Prognostic implications of BMP6 and OLFM1 in TCGA variation cohort in the Human Protein Atlas.
