# Supplementary material for: Low OLFM1 and BMP6 Expression Predicts Recurrence in Early-Stage Nonsquamous NSCLC with Pure Solid Tumor Appearance
Source: Cancer Res Commun. 2025 Dec 18;5(12):2186–96. doi: 10.1158/2767-9764.CRC-25-0186 (PMC12711631; doi:10.1158/2767-9764.CRC-25-0186)
Supplement: Supplementary Figure S9 — Figure S9. Prognostic implications of BMP6 and OLFM1 (stage-specific analysis). [file crc-25-0186_supplementary_figure_s9_suppsf9.pdf]

Supplementary Figure S9

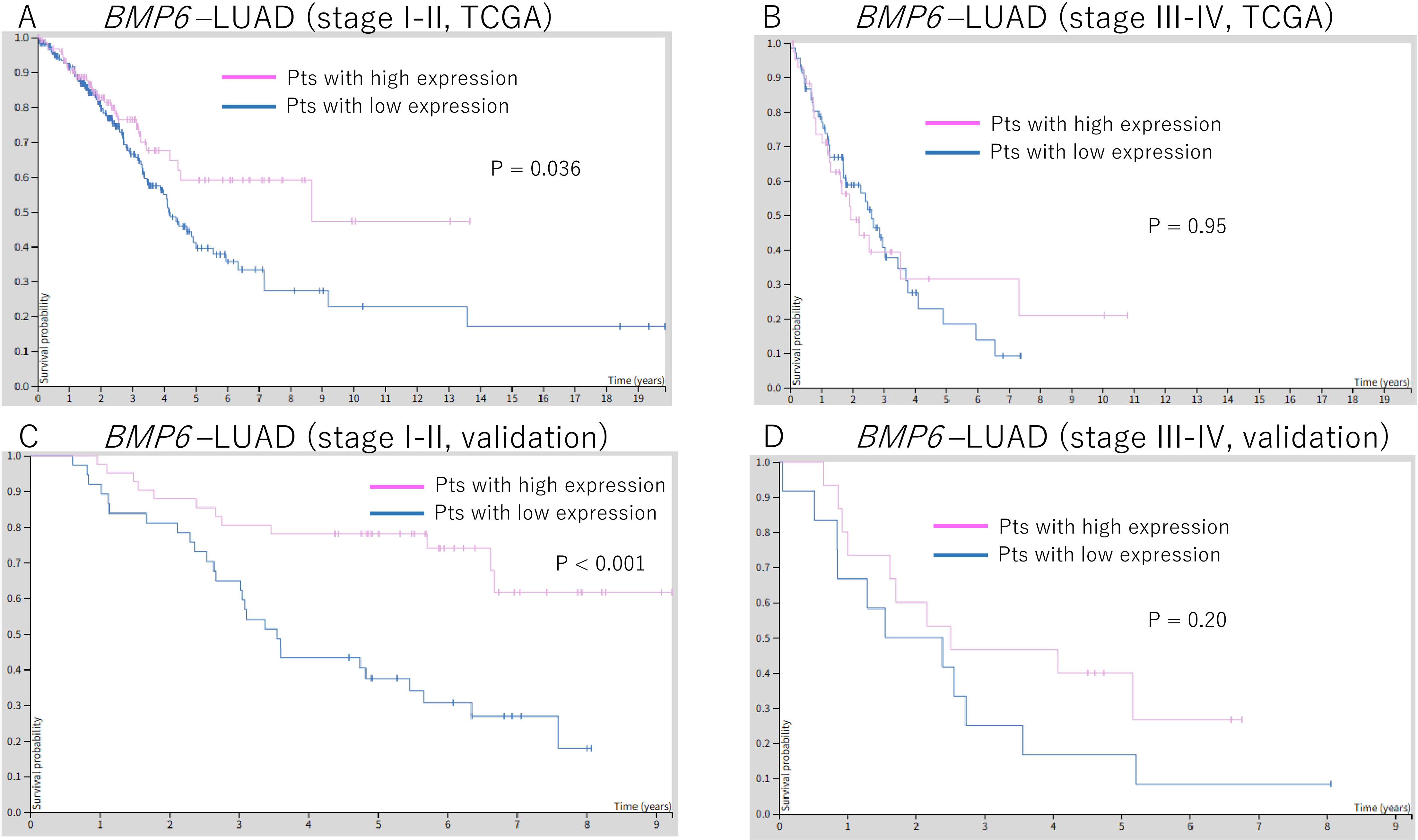

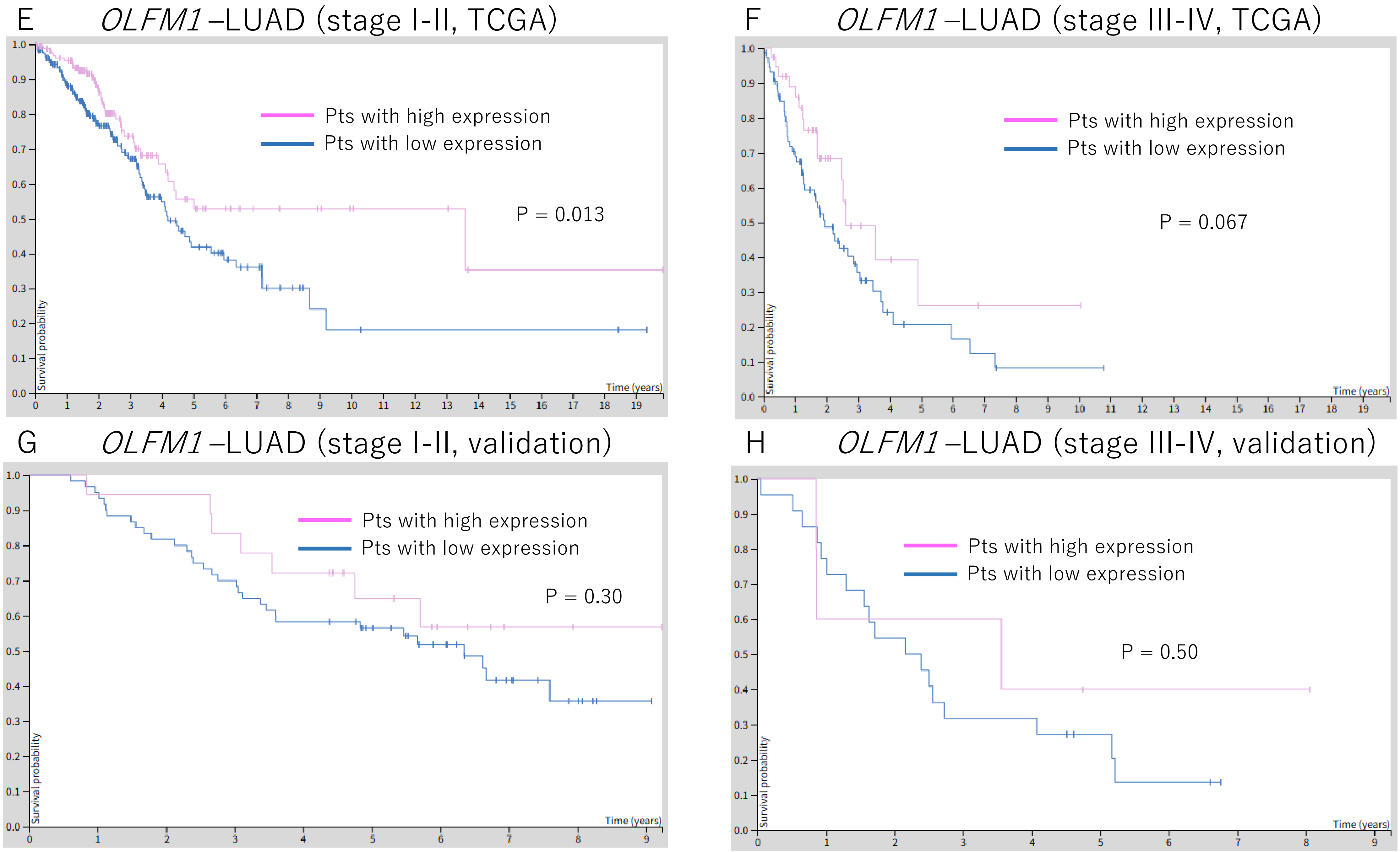

Supplementary Figure S9. Prognostic implications of BMP6 and OLFM1 (stage-specific analysis).
