# Supplementary material for: Low OLFM1 and BMP6 Expression Predicts Recurrence in Early-Stage Nonsquamous NSCLC with Pure Solid Tumor Appearance
Source: Cancer Res Commun. 2025 Dec 18;5(12):2186–96. doi: 10.1158/2767-9764.CRC-25-0186 (PMC12711631; doi:10.1158/2767-9764.CRC-25-0186)
Supplement: Supplementary Figure S10 — Figure S10. Overall survivals based on BMP6 and OLFM1 expression status in Cohort 2P (A) and in EGFR mutated cohort (B). [file crc-25-0186_supplementary_figure_s10_suppsf10.pdf]

Supplementary Figure S10

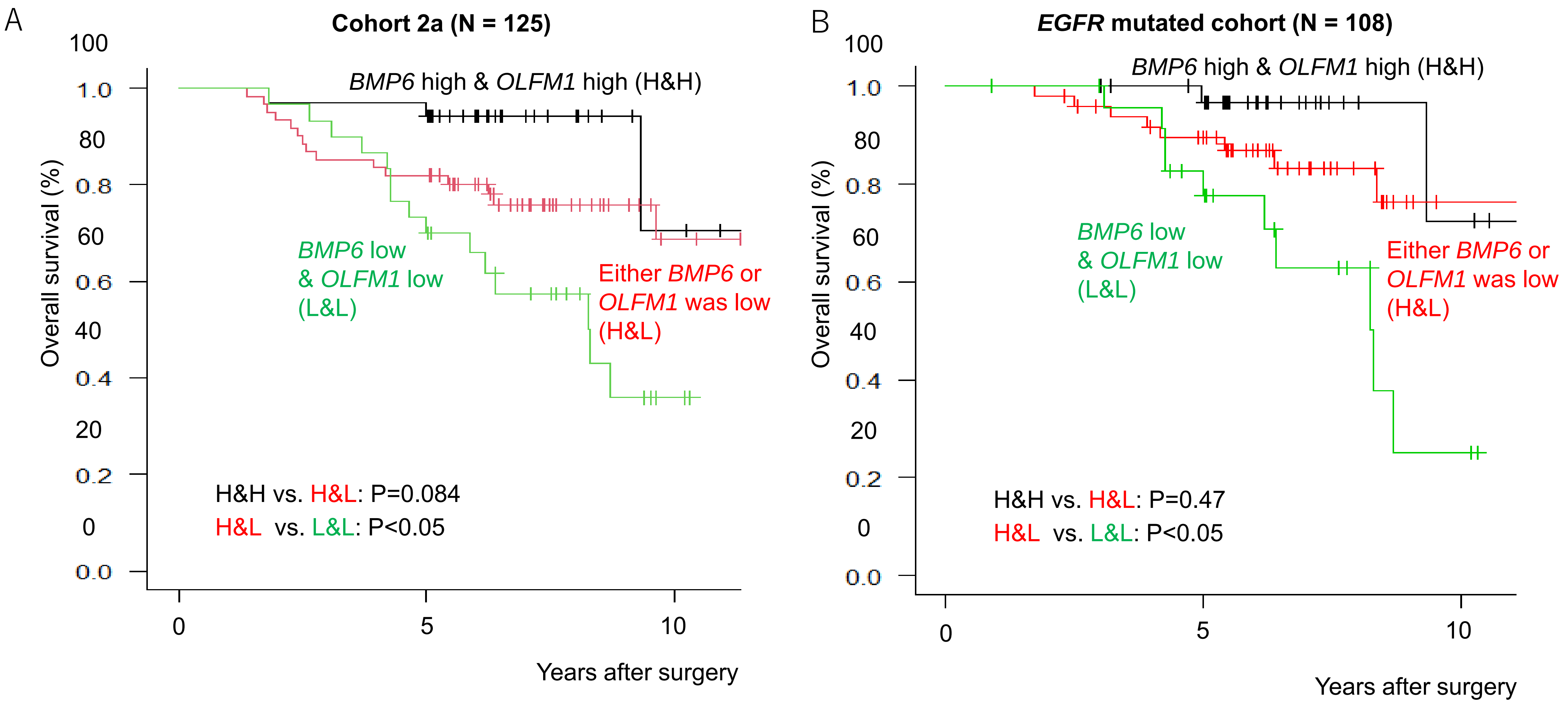

Supplementary Figure S10. Overall survivals based on *BMP6* and *OLFM1* expression status in Cohort 2a (A) and in *EGFR* mutated cohort (B).
