# Supplementary material for: Low OLFM1 and BMP6 Expression Predicts Recurrence in Early-Stage Nonsquamous NSCLC with Pure Solid Tumor Appearance
Source: Cancer Res Commun. 2025 Dec 18;5(12):2186–96. doi: 10.1158/2767-9764.CRC-25-0186 (PMC12711631; doi:10.1158/2767-9764.CRC-25-0186)
Supplement: Supplementary Figure S11 — Figure S11. Recurrence-free (A) and overall survivals (B) based on BMP6 and OLFM1 expression status in the entire Cohort 2. [file crc-25-0186_supplementary_figure_s11_suppsf11.pdf]

Supplementary Figure S11

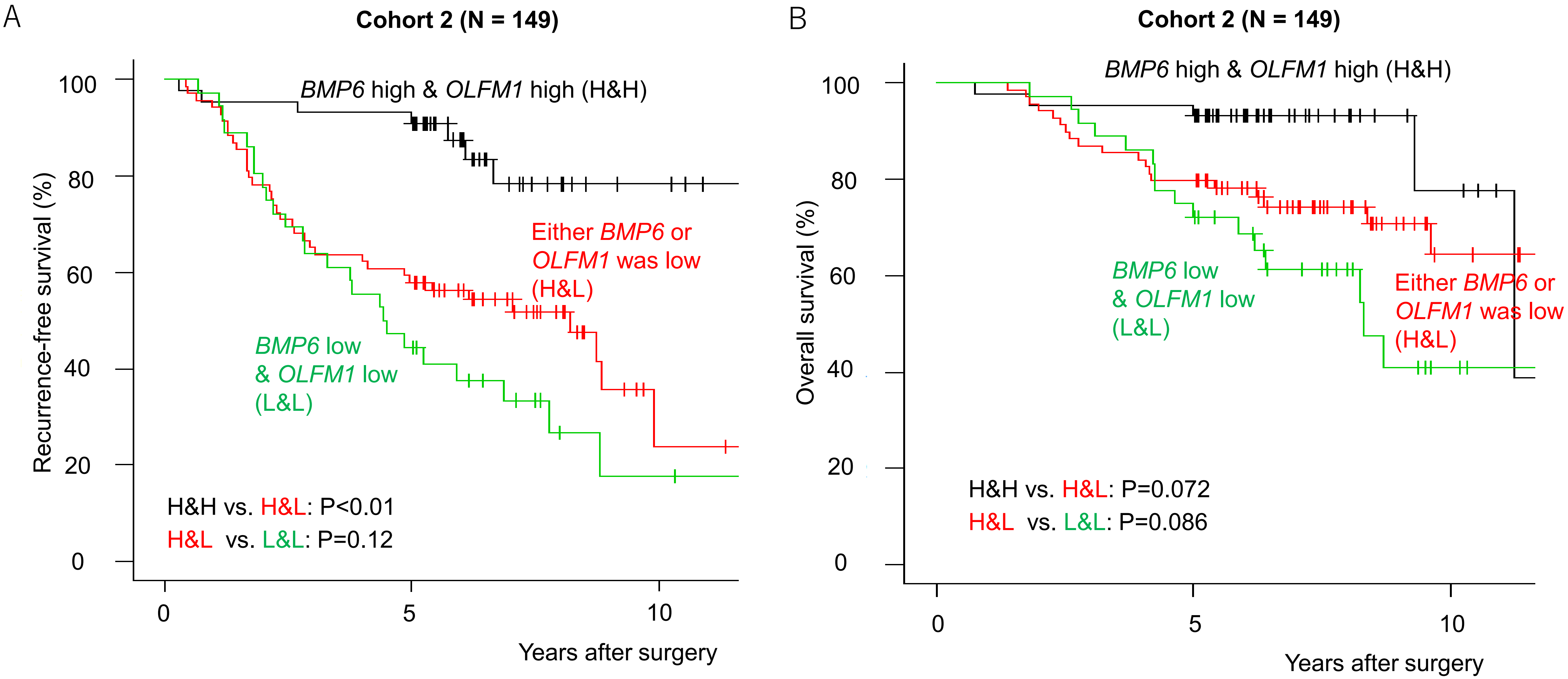

Supplementary Figure S11. Recurrence-free (A) and overall survivals (B) based on *BMP6* and *OLFM1* expression status in the entire Cohort 2.
