# Supplementary material for: Low OLFM1 and BMP6 Expression Predicts Recurrence in Early-Stage Nonsquamous NSCLC with Pure Solid Tumor Appearance
Source: Cancer Res Commun. 2025 Dec 18;5(12):2186–96. doi: 10.1158/2767-9764.CRC-25-0186 (PMC12711631; doi:10.1158/2767-9764.CRC-25-0186)
Supplement: Supplementary Table S1 — Table S1. Clinical characteristics of Patients (Cohort 1 and Cohort 2) [file crc-25-0186_supplementary_table_s1_suppst1.pdf]

Supplementary Table S1. Clinical characteristics of Patients (Cohort 1 and Cohort 2)

Factors	Cohort 1				Cohort 2 (N=149)
	Recurrence group（N=33）	Control group (N=33）	P-value	Std diff	
Sex (Female / Male)	17 (52%) / 16 (48%)	15 (45%) / 18 (55%)	0.81	0.12	94 (63%) / 55 (37%)
Age (≤65 / >66)	7 (21%) / 26 (79%)	5 (15%) / 28 (85%)	0.75	0.16	93 (62%) / 56 (38%)
Smoking (Yes/No)	20 (60%) / 13 (40%)	22 (67%) / 11 (33%)	0.80	0.12	55 (37%) / 94 (63%)
Median tumor size (25%-75%)	2.5（2.1 - 3.1)	2.5（1.9 - 3.2)	0.94	0.05	2.3 (1.8 - 3.0)
Pleural invasion (+ / -)	11 (33%) / 22 (67%)	10 (30%) / 23 (70%)	1.00	0.06	36 (24%) / 113 (76%)
