# Supplementary material for: Low OLFM1 and BMP6 Expression Predicts Recurrence in Early-Stage Nonsquamous NSCLC with Pure Solid Tumor Appearance
Source: Cancer Res Commun. 2025 Dec 18;5(12):2186–96. doi: 10.1158/2767-9764.CRC-25-0186 (PMC12711631; doi:10.1158/2767-9764.CRC-25-0186)
Supplement: Supplementary Table S2 — Table S2. The list of differently expressed genes in Cohort 1 [file crc-25-0186_supplementary_table_s2_suppst2.pdf]

Supplementary Table S2. The list of differently expressed genes in Cohort 1

class	Gene Names	Higher expression
N3	PCDH11X	Control group
N3	OR6K3	Control group
N3	MYH1	Control group
N3	CNTNAP4	Control group
N3	FAM187A	Control group
N3	SCN10A	Control group
N3	PXDNL	Control group
N3	PRPH	Control group
N3	C16orf96	Control group
N3	ASPRV1	Control group
N3	KCNK16	Control group
N3	GDF5−AS1	Control group
N3	KIR2DL4	Control group
N3	KCNK3	Control group
N3	PEG3	Control group
N3	LILRA1	Control group
N3	BCEL−TECTA	Control group
N3	CLEC17A	Control group
N3	CASP5	Control group
N3	LSMEM2	Control group
N3	FBN2	Control group
R3	ATP12A	Recurrence group
R3	FSD2	Recurrence group
R3	LOC100129175	Recurrence group
R3	LOC100132686	Recurrence group
R3	DSG3	Recurrence group
R3	FSIP2	Recurrence group
R3	LRRC19	Recurrence group
R3	UPK1B	Recurrence group
R3	TC3−AS1	Recurrence group
R3	CATSPERE	Recurrence group
R3	ANXA13	Recurrence group
R3	SYCP3	Recurrence group
R3	MIR6720	Recurrence group
R3	GDA	Recurrence group
R3	EDDM13	Recurrence group

R3	GNRHR	Recurrence group
R3	ONECUT1	Recurrence group
R3	ATP5MF−PTCD1	Recurrence group
R3	FIGNL2	Recurrence group
R3	PPM1N	Recurrence group
R3	PPP1R3G	Recurrence group
R3	ALKAL1	Recurrence group
R3	KIAA2012	Recurrence group
R3	TPTE2P5	Recurrence group
R3	CABCOCO1	Recurrence group
R3	VPS37D	Recurrence group
R3	GJB6	Recurrence group
R3	TAS2R43	Recurrence group
N2	PDE2A	Control group
N2	PTH1R	Control group
N2	SPIB	Control group
N2	PRAM1	Control group
N2	JAKMIP1	Control group
N2	TNFSF12−TNFSF13	Control group
N2	DNAJC5B	Control group
N2	BMP6	Control group
N2	RBP4	Control group
N2	SMIM41	Control group
N2	SYS1−DBNDD2	Control group
N2	STIMATE−MUSTN1	Control group
N2	CCL14	Control group
N2	LILRA5	Control group
N2	NFASC	Control group
R2	ARSD−AS1	Recurrence group
R2	EXOSC10−AS1	Recurrence group
R2	B3GNT6	Recurrence group
R2	PRKAR2B	Recurrence group
R2	EIF4EBP3	Recurrence group
R2	NECTIN3	Recurrence group
R2	MYEF2	Recurrence group
R2	MIR671	Recurrence group
N1	RPL39	Control group
N1	CXCL13	Control group
N1	NKG7	Control group

N1	HBA2	Control group
N1	CHIT1	Control group
N1	NCF1	Control group
N1	SLAMF7	Control group
N1	LOC100996842	Control group
N1	GZMH	Control group
N1	CPNE5	Control group
N1	DERL3	Control group
N1	GZMB	Control group
N1	TNXB	Control group
N1	OLFM1	Control group
N1	H3C2	Control group
N1	S100A9	Control group
N1	IGLL5	Control group
N1	H4C11	Control group
N1	H3C8	Control group
N1	PIM2	Control group
N1	PTGDS	Control group
R1	SPINK1	Recurrence group
R1	HPGD	Recurrence group
R1	PEG10	Recurrence group
R1	ZBTB41	Recurrence group
R1	CNIH4	Recurrence group
R1	TPT1	Recurrence group
R1	RPS3A	Recurrence group
R1	WDR74	Recurrence group
