# Supplementary material for: Low OLFM1 and BMP6 Expression Predicts Recurrence in Early-Stage Nonsquamous NSCLC with Pure Solid Tumor Appearance
Source: Cancer Res Commun. 2025 Dec 18;5(12):2186–96. doi: 10.1158/2767-9764.CRC-25-0186 (PMC12711631; doi:10.1158/2767-9764.CRC-25-0186)
Supplement: Supplementary Table S3 — Table S3. Clinical characteristics of Patients [file crc-25-0186_supplementary_table_s3_suppst3.pdf]

Supplementary Table S3. Clinical characteristics of Patients (Cohort 2 PolyA sequencing group; Cohort 2a)

Factors	Cohort 2a (N=125)
Sex (Female / Male)	81 (65%) / 44 (35%)
Age (≤65 / >66)	77 (62%) / 48 (38%)
Smoking (Yes/No)	44 (35%) / 81 (65%)
Median tumor size (25%-75%)	2.4 (1.9 - 3.2)
Pleural invasion (+ / -)	27 (22%) / 98 (78%)
