# Supplementary material for: Low OLFM1 and BMP6 Expression Predicts Recurrence in Early-Stage Nonsquamous NSCLC with Pure Solid Tumor Appearance
Source: Cancer Res Commun. 2025 Dec 18;5(12):2186–96. doi: 10.1158/2767-9764.CRC-25-0186 (PMC12711631; doi:10.1158/2767-9764.CRC-25-0186)
Supplement: Supplementary Table S4 — Table S4. Multivariable analysis of 6 genes for RFS in the Cohort 2a (N = 125) [file crc-25-0186_supplementary_table_s4_suppst4.pdf]

Supplementary Table S4. Multivariable analysis of 6 genes for RFS in the Cohort 2a (N = 125)

Factors	HR	95% CI	p-value
BMP6	1.89	1.06 - 3.11	**0.029**
KCNK3	1.44	0.69-2.97	0.33
NFASC	1.13	0.61-2.09	0.69
OLFM1	2.44	1.24-4.81	**<0.01**
PEG3	1.65	0.88-3.08	0.12
TNXB	1.13	0.61-2.07	0.70
